# Response Interruption and Redirection for Stereotypy: A Quality Review and Ethical Considerations

**DOI:** 10.1177/01454455261434871

**Published:** 2026-04-29

**Authors:** Hannah MacNaul, Anh Nguyen

**Affiliations:** 1 University of Texas at San Antonio, USA; 2 University of South Florida, USA

**Keywords:** response interruption and redirection (RIRD), stereotypy, autism, methodological quality, neurodiversity-affirming practice

## Abstract

Response interruption and redirection (RIRD) is a commonly evaluated intervention for reducing stereotypy, a core feature of autism spectrum disorder (ASD). Although RIRD has been recognized as an evidence-based practice, increasing emphasis on neurodiversity affirming and contextually grounded intervention has highlighted the need to reexamine both the methodological rigor of the literature and the conditions under which RIRD is appropriate. This systematic review evaluated the quality and ethical contextualization of published RIRD studies by applying the What Works Clearinghouse (WWC) single case design standards and an ethical decision framework informed by the National Institutes of Health guiding principles and contextual fit literature. A total of 42 articles met inclusion criteria, yielding 277 cases. Of these, approximately 9% met WWC design standards without reservations, 44% met standards with reservations, and 47% did not meet standards. Of the 144 cases eligible for evidence review, 67% demonstrated strong or moderate evidence of a functional relation. Ethical coding showed that most participants (76%) had an explicit rationale for treatment, yet reporting of procedural fidelity and social validity remained inconsistent. Only 38% of articles assessed social validity. Among those that did, stakeholder ratings were consistently positive, and participant-based measures indicated that when given a choice, individuals often preferred RIRD over alternatives. Taken together, findings suggest that RIRD can be effective and ethically defensible when implemented following established prerequisites such as contextual fit, functional behavior assessment, high fidelity implementation, and consideration of client preferences. Implications for practice and recommendations for designing neurodiversity affirming, high quality RIRD research are discussed.

**Public Significance Statement:**

Stereotypic behaviors, such as hand-flapping or vocal repetition, are an important part of many autistic individuals' experiences. While reducing stereotypy can sometimes help with learning or social access, it is essential that interventions respect each person's needs and preferences. This review examined the strength and contextual appropriateness of research supporting response interruption and redirection (RIRD), an intervention sometimes used to reduce stereotypy. Findings show that while RIRD can be helpful, the quality of research varies, and the procedure should only be used when clearly justified, preferred, and implemented with care. This means that careful, individualized decision-making is needed when considering RIRD to ensure it supports, rather than suppresses, the unique ways autistic individuals communicate and engage with the world.

## Introduction

Restricted and repetitive behaviors are a defining characteristic of autism spectrum disorder (ASD; American Psychiatric Association, 2013). Among these, stereotypy, defined as repetitive vocalizations, motor movements, or object manipulations, has historically been conceptualized within applied behavior analysis (ABA) as behavior that may interfere with learning, social engagement, and broader participation in community settings (Healy & Leader, 2011; Rapp & Vollmer, 2004). As the neurodiversity movement has gained momentum, however, autistic individuals and advocates have emphasized that stereotypy often serves meaningful functions, such as emotional regulation, communication, or expressions of joy (Kapp, 2020). In this context, interventions aimed at reducing stereotypy have faced increased scrutiny, particularly when efforts are perceived as pathologizing harmless behaviors or prioritizing normative appearance over individual autonomy (Bottema-Beutel et al., 2021). Consequently, behavior analysts are challenged to ensure that intervention efforts are ethically grounded, contextually justified, and aligned with the expressed values and preferences of the individuals they serve.

Despite evolving ethical considerations, there remain contexts in which reducing stereotypy may be warranted to support safety, learning, or quality of life. For example, stereotypic behavior that disrupts academic engagement, impedes the development of social relationships, or presents health risks may limit access to valued environments and opportunities (Koegel et al., 2012; Steinbrenner et al., 2020). In such cases, thoughtfully applied interventions that are sensitive to the contextual function and meaning of stereotypy can serve as an important component of individualized, person-centered support plans. Thus, research into interventions for stereotypy continues to be a critical area of inquiry within behavior analysis, provided that interventions are implemented in ways that affirm the autonomy and dignity of autistic individuals.

Response interruption and redirection (RIRD) is one such intervention that has received considerable empirical attention. RIRD involves interrupting stereotypic behavior contingent upon its occurrence and redirecting the individual to engage in alternative, appropriate behaviors, often through motor or vocal tasks (Ahearn et al., 2007). Early work showed that RIRD functions as a punishment-based procedure, with decreases in stereotypy produced by contingent interruption and effort regardless of the specific form or type of redirection task used (Ahrens et al., 2011). Although initially described as a relatively uniform procedure, subsequent research has revealed considerable variability in how RIRD is operationalized across studies. Procedural variables include the type of redirection task (e.g., motor versus vocal), the complexity or mastery level of the tasks, the prompting procedures used, and the criteria for terminating RIRD sequences. In addition, RIRD has sometimes been combined with other interventions, such as differential reinforcement of alternative behavior (DRA) or response cost procedures, to enhance effectiveness or promote generalization. This heterogeneity underscores the importance of not treating RIRD as a singular, fixed intervention but rather as a flexible framework that can be adapted depending on contextual variables and participant characteristics. However, because the procedure operates through punishment and can take multiple forms, the quality of the evidence supporting these variations becomes especially important.

The National Clearinghouse on Autism Evidence and Practice (NCAEP; Steinbrenner et al., 2020) identified RIRD as an evidence-based practice, citing multiple single-case design studies demonstrating its effectiveness in reducing stereotypy across diverse populations. However, designation as an evidence-based practice primarily reflects demonstrations of intervention effectiveness, rather than comprehensive assessments of methodological quality or contextual appropriateness. Subsequent reviews have followed this pattern. Spencer and Alkhanji (2018) synthesized RIRD studies but did not evaluate internal validity or risk of bias, and Lydon et al. (2013) compared RIRD to redirection without assessing methodological rigor. When quality measures were incorporated, the approaches differed. For example, Ledford et al. (2023) evaluated stereotypy interventions using the Single Case Analysis and Review Framework (SCARF; Ledford et al., 2016/2023), which provides a structured method for examining selected indicators of internal and external validity but does not align with the more frequently used What Works Clearinghouse single case design standards (WWC, 2022). As a result, the current review extends prior quality evaluations by applying the widely utilized What Works Clearinghouse single case design standards (WWC, 2022), offering a complementary perspective on methodological rigor within the RIRD literature.

In addition to evaluating methodological quality, an ethical analysis of RIRD requires grounding in established frameworks that clarify when and how interventions should be considered appropriate. The ethical variables used in the present review draw primarily from three complementary sources. First, the National Institutes of Health (NIH) provides guiding principles for ethical research that emphasize the importance of social and clinical value, risk minimization, fair participant selection, and scientific validity (NIH, n.d.). These principles offer a foundational structure for determining whether an intervention is necessary, justified, and implemented in ways that protect participant welfare. Second, contextual fit indicators identified by Rojas et al. (2025) highlight the importance of treatment need, efficiency, stakeholder preference, and cultural relevance, which are essential for understanding the real-world appropriateness of RIRD in diverse settings. Third, trends identified by Ferrier et al. (2025) underscore the evolving role of RIRD within punishment-based interventions and reinforce the need to ensure that procedures involving contingent effort are accompanied by clear rationales, acceptable levels of intrusiveness, and evidence of social validity. Together, these sources provide a comprehensive and ethically informed framework for examining how RIRD has been justified and implemented across the literature and for assessing whether its use aligns with contemporary expectations for responsible, person-centered practice. Accordingly, the present review examines the conditions under which RIRD has been evaluated for reducing stereotypy, with particular attention to whether treatment need is clearly articulated, functional behavior assessment results support intervention selection, methodological rigor is established, and procedural fidelity and social validity are transparently reported.

The purpose of the current review was to systematically evaluate the RIRD literature using both methodological and ethical criteria. Specifically, we applied the What Works Clearinghouse single case design standards (WWC, 2022) to assess the rigor of existing studies and coded a set of ethical variables derived from NIH guiding principles, contextual fit frameworks, and recent analyses of punishment-based interventions. By integrating these complementary perspectives, the review aims to clarify the strengths and limitations of the evidence supporting RIRD and to offer guidance for its responsible and contextually appropriate use in practice.

## Methods

### Literature Search Procedures

**Figure 1.**
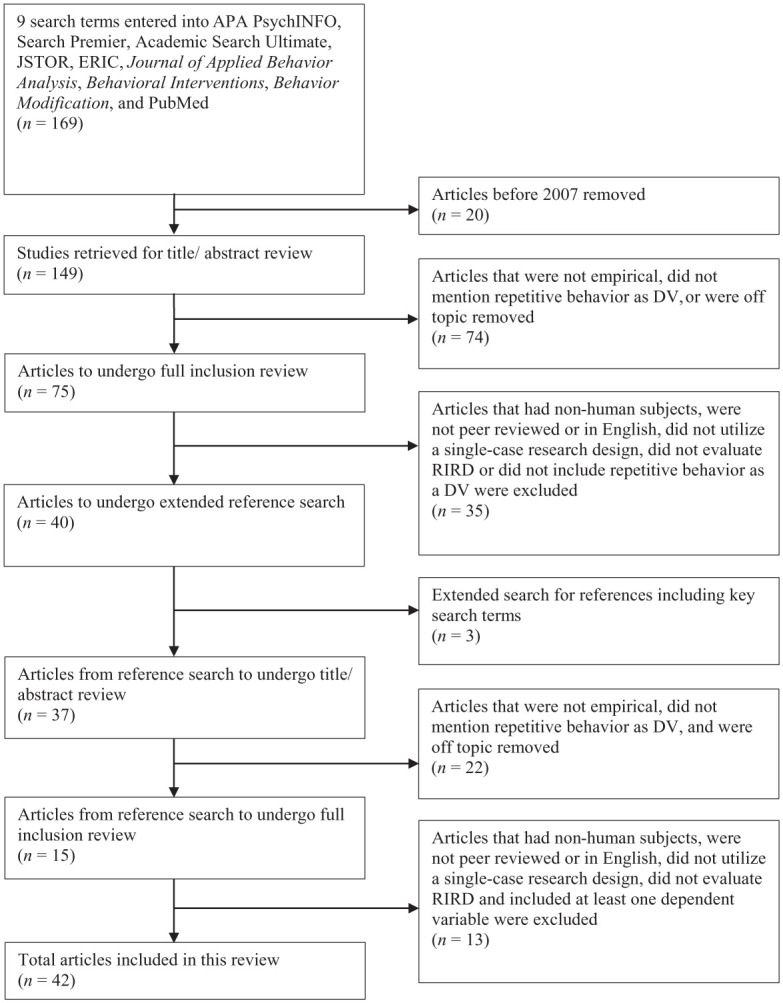
Search figure.

This review was registered in PROSPERO (CRD420251177814) and reported in accordance with the Preferred Reporting Items for Systematic Reviews and Meta Analyses (PRISMA) 2020 guidelines. We searched four databases and three behavior analytic journals to identify potential studies for this review, including APA PsycINFO, Academic Search Premier, JSTOR, ERIC, *Journal of Applied Behavior Analysis*, *Behavioral Interventions*, and *Behavior Modification*. In December 2023, we initiated the first search and restricted the search to articles published since the Ahearn et al. (2007) to December 2023. Results were limited to English-language and peer-reviewed research. All databases and journals were searched by combining the terms, “automatic reinforcement” and “stereotypy” with the terms, “redirection,” “response redirection,” “response interruption and redirection,” “RIRD,” “contingent demands,” and “overcorrection.” These search procedures yielded a total of 116 articles. The titles and abstracts of the 116 articles were screened to identify articles for potential inclusion in this review. During the title-abstract review, articles were excluded if they (a) were not empirical (e.g., case studies, reviews, commentary), (b) indicated the dependent variable was a behavior other than stereotypic behavior (e.g., aggression, SIB), or (c) were off topic to the study of interest (e.g., token economies, picture activity schedules, aneurisms). Following the title-abstract review, 75 articles were identified for further review. To ensure the review remained up to date, we re-ran the full search in May 2025 using the same databases, journals, and search parameters. No additional articles meeting inclusion criteria were identified (Figure 1).

### Inclusion Criteria

The 75 articles were then downloaded and evaluated based on preset inclusion criteria. To be included in the literature review, an article had to meet the following criteria: (a) participants were human subjects, (b) peer-reviewed and published in English, (c) used a single-case research design, (d) evaluated RIRD, procedurally defined as interruption of the repetitive behavior and redirection to other behavior contingent on repetitive behavior, alone or in combination with other intervention procedures (e.g., response cost) and (e) measured at least one dependent variable pertaining to repetitive behavior. Regarding the RIRD procedural definition, articles were included if the intervention(s) included redirection to alternative and/or appropriate behavior (e.g., clap your hands, hands down, hand in lap, touch your head). After the application of these criteria, a total of 40 studies met the inclusion criteria. An extended search was conducted by reviewing the references of each of these 40 articles. Articles identified during the extended search were reviewed using the same procedures as described above. Of the 37 additional articles identified via the extended search, 15 were selected for the full inclusion review, and two studies met the inclusion criteria. At the end of the search, a total of 42 articles were included in the current review.

### Application of What Works Clearinghouse Standards

All included studies were evaluated using the What Works Clearinghouse single case design standards (WWC, 2022). Each study was coded separately for WWC design standards and evidence analyses. WWC design standards assess whether the study demonstrates sufficient methodological rigor, including systematic manipulation of the independent variable, adequate measurement procedures, acceptable levels of interobserver agreement, evidence of at least three attempts to demonstrate an effect, and adequate data points per phase. Full descriptions of each WWC criterion and decision rule are detailed in the Supplemental Materials.

Coding was conducted at the level of the individual case, with each participant by condition combination considered a separate case. For example, in Cividini-Motta et al. (2019), the participant Sansita contributed six cases due to multiple intervention comparisons and dependent variables. Across the 42 included studies, 109 participants produced a total of 277 cases. Following WWC procedures, each case received an overall design rating of Meets Standards, Meets Standards with Reservations, or Does Not Meet Standards. Only cases rated as Meets Standards or Meets Standards with Reservations were retained for WWC evidence coding and for inclusion in the descriptive synthesis.

For studies that met design standards, WWC evidence standards were then applied to evaluate whether the study demonstrated a functional relation. Evidence ratings were assigned at the level of the individual case, defined as a single participant by dependent variable combination. When multiple dependent variables were evaluated for a given participant, each dependent variable was treated as a separate case and coded independently for design and evidence standards. Table 1 aggregates cases that received identical WWC design and evidence ratings for reporting efficiency only and does not reflect combined analysis of multiple dependent variables within a single case. Evidence coding followed WWC visual analysis guidelines, which integrate evaluation of baseline patterns, between phase changes (level, trend, variability, overlap, immediacy), and within phase consistency. Each case was assigned an overall evidence rating of Strong Evidence, Moderate Evidence, or No Evidence of a causal relation. Full coding procedures and scoring rubrics for evidence standards are also provided in the Supplemental Materials.

**Table 1.** Descriptive Synthesis Results.

Article	Name	Age	Sex	Diagnosis	Verbal repertoire	Communication modality	Verbal complexity	Target behavior	Contextual fit	FBA	RIRD	Social validity
Ahearn et al. (2007)	Mitch	3	M	Other	Yes	Vocal	Simple	VS	Yes	FA	RIRD	No
	Nicki	7	F	ASD	Yes	Vocal	Simple	VS	Yes	FA	RIRD	
	Peter	11	M	ASD	Yes	Vocal	Simple	VS	Yes	FA	RIRD	
	Alice	7	F	ASD	Yes	Picture exchange, Sign / Gesture	Simple	VS	Yes	FA	RIRD	
Ahrens et al. (2011)	Hal	4	M	ASD	Yes	Vocal	Simple	VS	Yes	FA	RIRD	No
	Bobby	6	M	ASD	Yes	Vocal	Complex	VS	Yes	FA	RIRD	
	David	4	M	ASD	Yes	Vocal	Simple	VS, MS	Yes	FA	RIRD	
	Glen	5	M	ASD	Yes	Vocal	Complex	VS, MS	Yes	FA	RIRD	
Barszcz et al. (2021)	Abby	5.1	F	ASD	Yes	Vocal	NR	VS	No	NR	RIRD	No
	Ben	4.8	M	ASD	Yes	Vocal	NR	VS	No	NR	RIRD	
	Carol	8	F	ASD	Yes	Vocal	NR	VS	No	NR	RIRD	
	David	5.7	M	ASD	Yes	Vocal	NR	VS	No	NR	RIRD	
Brusa and Richman (2008)	Mark	8	M	ASD	Yes	Vocal	Complex	MS	Yes	FA	VR + RB	No
Callahan et al. (2023)	Kevin	6	M	ASD	Yes	NR	NR	VS, MS	Yes	NR	RIRD	Yes; Survey by BCBA
	Joe	7	M	ASD	Yes	NR	NR	VS, MS	Yes	NR	RIRD	
	Nick	3	M	ASD	Yes	NR	NR	VS, MS	Yes	NR	RIRD	
Carroll and Kodak (2014)	Parker	5	M	ASD	Yes	Vocal	Complex	VS	No	NR	RIRD	No
	Will	8	M	ASD	Yes	Vocal	Complex	VS	Yes	NR	RIRD	
Cassella et al. (2011)	Adam	4	M	ASD	Yes	NR	NA	VS	Yes	Indirect	RIRD	Yes; Survey from Caregivers
	Chris	7	M	ASD	Yes	NR	NA	VS	Yes	Indirect	RIRD	
Chen and Traub (2022)	Eric	30	M	ASD	Yes	Vocal	Complex	VS	Yes	NR	RIRD	No
Cividini-Motta et al. (2019)	Ariel	5	F	ASD	Yes	Vocal	Complex	MS	No	Automatic Screening	RIRD, RIRD + DRA	Yes, Concurrent-chain preference assessment with participants
	Sansita	5	F	ASD	Yes	Sign / Gesture, Vocal	Simple	MS	No	Automatic Screening	RIRD, RIRD + DRA	
	Lewis	8	M	ASD	Yes	Sign / Gesture	Simple	VS	Yes	Automatic Screening	RIRD, RIRD + DRA	
Cividini-Motta et al. (2020)	Eddie	13	M	ASD	Yes	Vocal	Simple	Public Masturbation	Yes	Automatic Screening	RIRD	Yes; Survey with Individuals who interacted with participants regularly
	David	20	M	ASD	Yes	Vocal	Complex	Public Masturbation	Yes	Automatic Screening	RIRD	
	Carlos	13	M	ASD	Yes	SGD	Simple	Public Masturbation	Yes	Automatic Screening	RIRD	
	Emily	6	F	ASD	Yes	Vocal	Complex	Public Masturbation	Yes	FA	RIRD	
Colón and Ahearn (2019)	Chad	15	M	ASD	Yes	Vocal, Picture exchange	Simple	VS	Yes	FA	RIRD	No
	Morris	14	M	ASD	Yes	Vocal	Simple	VS	Yes	FA	RIRD	
	Noah	15	M	ASD	Yes	Vocal	Complex	VS	Yes	FA	RIRD	
	Cora	16	F	ASD	Yes	Vocal	Simple	VS	Yes	FA	RIRD	
	Kent	21	M	ASD	Yes	SGD	NR	VS	Yes	FA	RIRD	
Colón et al. (2012)	Anna	8	F	ASD	Yes	Vocal	Simple	VS	Yes	FA	RIRD	No
	Parker	10	M	ASD	Yes	Vocal	Complex	VS	Yes	FA	RIRD	
	Jeff	10	M	ASD	Yes	Vocal	Simple	VS	Yes	FA	RIRD	
Cook and Rapp (2020)	Sam	7	M	ASD	NR	NR	NA	VS, MS	Yes	Automatic Screening	PP OC	
DeRosa et al. (2019)	Zane	6	M	ASD	Yes	Vocal	Simple	MS	No	FA	RIRD	No
	Caden	9	M	ASD	Yes	Vocal	Complex	MS	No	FA	RIRD	
	Richard	19	M	ASD	Yes	Sign / Gesture	Simple	MS	No	FA	RIRD	
Dickman et al. (2012)	Tobias	5.6	M	Other	Yes	NR	NA	VS	No	FA	RIRD, RIRD + DRI	No
Falligant and Dommestrup (2020)	David	12	M	ASD	Yes	NR	NA	MS	Yes	FA	RIRD	No
Frewing et al. (2015)	John	19	M	ASD	Yes	Vocal	Complex	VS, MS, Other	No	NR	RIRD	Yes; Participant Heart Rate
Gauthier et al. (2020)	Mary	8	F	ASD	Yes	Vocal	Complex	VS	Yes	FA	RIRD	No
	Matt	14	M	ASD	Yes	Picture exchange, Sign / Gesture	Simple	VS	Yes	FA	RIRD	
	Ben	16	M	ASD	Yes	SGD	NR	VS	Yes	FA	RIRD	
	Steve	14	F	ASD	Yes	Vocal	Complex	VS	Yes	FA	RIRD	
Gibbs et al. (2018)	Elizabeth	4	F	ASD	Yes	Vocal	Complex	VS	Yes	FA	MS + RIRD	Yes; Caregiver Survey
	Matthew	7	M	ASD	Yes	Vocal	Complex	VS	Yes	FA	MS + RIRD	
Gibney et al. (2020)	Ben	16	M	ASD	Yes	Vocal	Complex	VS	Yes	FA	RIRD	Yes; Survey with Parents and Teachers
	Andy	16	M	ASD	Yes	Vocal	Simple	VS	Yes	FA	RIRD	
	Kallum	14	M	ASD	Yes	Vocal	Complex	VS	Yes	FA	RIRD	
	Harriet	12	M	ASD	Yes	Vocal	Simple	VS	Yes	FA	RIRD	
A. F. Giles et al. (2012)	Kelly	10	F	ASD	Yes	Sign / Gesture	Simple	MS	No	FA	RD, RB	Yes; Concurrent chain preference assessment with participants
	Adam	6	M	ASD	Yes	Sign / Gesture	Simple	MS	No	FA	RD, RB	
	Spike	10	M	ASD	Yes	Vocal	Complex	MS	No	FA	RD, RB	
A. Giles et al. (2018)	James	12	M	ASD	Yes	Vocal	Complex	MS	Yes	NA	RIRD	Yes; Survey with Teaching Assistants
	Tim	9	M	ASD	Yes	Picture exchange	Simple	MS	Yes	NA	RIRD	
	Daniel	6	M	ASD	Yes	Picture exchange	Simple	MS	Yes	NA	RIRD	
Gould et al. (2019)	David	10	M	ASD	Yes	Sign / Gesture	Simple	MS	Yes	Automatic Screening	RIRD	No
Liu-Gitz and Banda (2010)	Dylan	10	M	ASD	Yes	NR	NA	VS	Yes	FA	RIRD	Yes; Survey with Teacher
Love et al. (2012)	Ivan	8	M	ASD	Yes	Vocal	Complex	VS	Yes	FA	RIRD, MS + RIRD	Yes; Survey with Caregivers
	Troy	9	M	ASD	Yes	Vocal	Complex	VS	Yes	FA	RIRD, MS + RIRD	
Martinez et al. (2016)	Peter	5	M	ASD	Yes	Vocal	Complex	VS	No	Descriptive Assessment	RIRD	No
McNamara and Cividini-Motta (2019)	Karl	9	M	ASD	Yes	Vocal	Complex	VS	Yes	FA	RIRD, RIRD + RC	Yes; Survey with Caregivers
	Sammy	8	M	ASD	Yes	Vocal	Simple	VS	Yes	FA	RIRD, RIRD + RC	
	Jon	11	M	ASD	Yes	Vocal	Complex	VS	Yes	FA	RIRD, RIRD + RC	
Meany-Daboul et al. (2007)	Amy	7	F	ASD	No	NR	NA	VS	No	FA	RIRD	No
	Daniel	21	M	ASD	No	NR	NA	VS	No	FA	RIRD	
	Beth	14	F	ASD	No	NR	N	MS	No	FA	RIRD	
Miguel et al. (2009)	James	4	M	ASD	Yes	NR	NA	VS	Yes	FA	RIRD, Other	No
Pastrana et al. (2013)	Emmett	10	M	ASD, Down Syndrome	No	NA	NA	MS	No	Automatic Screening	RIRD	No
	Andrew	7	M	ASD	No	NA	NA	MS	No	Automatic Screening	RIRD	
Peters and Thompson (2013)	Max	17	M	ADHD, Other	Yes	Vocal, SGD	Complex	MS	Yes	FA	PP OC	No
	Wes	9	M	ASD	Yes	Vocal	Complex	MS	Yes	FA	PP OC	
	Brett	24	M	ASD	Yes	Sign / Gesture	Simple	MS	Yes	FA	PP OC	
Saini et al. (2015)	Fabian	5	M	ASD	Yes	Vocal, Picture exchange	Simple	MS	Yes	FA	RIRD	No
	Walter	8	M	ASD	Yes	Vocal	Complex	VS	Yes	FA	RIRD	
	Carlton	5	M	ASD	Yes	Vocal	Complex	MS	Yes	FA	RIRD	
	Barry	5	M	ASD	Yes	Vocal	Complex	MS	Yes	FA	RIRD	
Schumacher and Rapp (2011)	Miranda	5	F	ASD	Yes	Vocal	Complex	VS	No	Automatic Screening	RIRD	No
	Nick	8	M	ASD	Yes	Vocal	Complex	VS	No	Automatic Screening	RIRD	
Scully et al. (2023)	Ben	.8	M	Other	NR	NR	NA	MS	Yes	NR	RIRD	No
Shawler et al. (2020)	Paul	7	M	ASD	Yes	Vocal	Complex	VS	Yes	Automatic Screening	RIRD	Yes; Survey with Caregivers
	Jane	5	F	ASD	Yes	Vocal	Complex	VS	Yes	Automatic Screening	RIRD	
Shawler and Miguel (2015)	Sally	5	F	ASD	Yes	Vocal	NR	VS	Yes	FA	RIRD	Yes; Survey with Caregivers
	Charlie	12	M	ASD	Yes	Vocal	Complex	VS	Yes	FA	RIRD	
	Josh	7	M	ASD	Yes	Vocal	Complex	VS	Yes	FA	RIRD	
	Adam	6	M	ASD	Yes	Vocal	Simple	VS	Yes	FA	RIRD	
	Brayden	12	M	ASD	Yes	Vocal	Complex	VS	Yes	FA	RIRD	
Sivaraman and Rapp (2020)	Arun	7	M	ASD	Yes	Vocal	Complex	VS	No	FA	RIRD	No
	Jason	5	M	ASD	Yes	Vocal	Simple	VS	No	FA	RIRD	
Sloman et al. (2017)	Elliot	13	M	ASD	Yes	Vocal	Complex	VS	No	FA	RIRD	Yes; Survey with Staff Implementers
Steinhauser et al. (2021)	Andy	12	M	ASD	Yes	Vocal	Simple	VS, MS	Yes	FA	DRA + C-RD	No
	Hank	20	M	ASD	Yes	SGD	NR	MS	Yes	FA	DRA + C-RD	
	Scott	18	M	ASD	Yes	Vocal	Simple	VS, MS	Yes	FA	DRA + C-RD	
	Seth	14	M	ASD	Yes	Vocal	Complex	VS, MS	Yes	FA	DRA + C-RD	
	Sam	14	M	ASD	Yes	Vocal	Complex	VS	Yes	FA	DRA + C-RD	
Toper-Korkmaz et al. (2018)	Nancy	6	F	ASD	Yes	Vocal	Simple	VS	Yes	Automatic Screening	RIRD	No
	Areli	6	F	ASD	Yes	Vocal	Complex	VS	Yes	Automatic Screening	RIRD	
	Bryan	4	M	ASD	Yes	Vocal	Complex	VS	Yes	Automatic Screening	RIRD	
Wells et al. (2016)	Roger	13	M	ASD	Yes	Vocal	Complex	VS	Yes	FA	RIRD	Yes; Interview with Teachers and Educational Assistants
Wunderlich and Vollmer (2015)	Ariel	12	F	ASD	Yes	Vocal, Sign / Gesture	Simple	VS	Yes	FA	RIRD	No
	Harold	6	M	ASD	Yes	Sign / Gesture	Simple	VS	Yes	FA	RIRD	
	Kora	15	F	Other	No	NR	NA	VS	Yes	FA	RIRD	
	Abby	11	F	ASD	Yes	Vocal, SGD	Complex	VS	Yes	FA	RIRD	
	Daisy	5	F	ASD	Yes	Vocal	Complex	VS	Yes	FA	RIRD	
	Drake	20	M	ASD	Yes	Sign / Gesture	Simple	VS	Yes	FA	RIRD	
	Camilla	4	F	ASD	Yes	Vocal, Sign / Gesture	Simple	VS	Yes	FA	RIRD	

*Note*. ASD = autism spectrum disorder; C-RD = contingent redirection; DRA = differential reinforcement of alternative behavior; FA = functional analysis; F = female; M = male; NA = not applicable; NR = not reported; PPOC = positive practice overcorrection; RB = response blocking; RC = response cost; RIRD = response interruption and redirection; SGD = speech generating device.

### Data Extraction Procedures

Two researchers independently extracted descriptive information from all studies that met WWC design standards using a structured coding system. Extracted variables were organized at the participant level and included demographic characteristics (age, sex, diagnoses), communication repertoire, contextual fit indicators, functional behavior assessment procedures, and RIRD intervention characteristics. Communication repertoire was coded using author descriptions of verbal complexity (simple, complex, not reported), communication modality (vocal verbal, manual sign, gesture, or SGD), and any additional detail provided about expressive or receptive skills. If authors reported that participants used a speech generating device (SGD) or sign language but did not specify criteria for simple or complex verbal skills, verbal complexity was coded as not reported (NR). When verbal repertoires were described only in group level inclusion criteria rather than individually, those criteria were used to code participants’ verbal characteristics.

Contextual fit indicators included whether authors described concerns from caregivers or stakeholders, barriers related to learning or social access, or other rationale for targeting stereotypy; these rationales were extracted verbatim when available. Functional behavior assessment procedures were coded as indirect, descriptive, or experimental (for example, automatic-only screenings or full functional analysis (FA)). RIRD characteristics were extracted in detail, including implementation setting (controlled or naturalistic), implementer, topography of redirection tasks (motor or vocal), mastery level of tasks (mastered or non-mastered), redirection topography (verbal or physical), prompting procedures (for example, representation, progressive prompt hierarchy, verbal or physical prompts), and termination criteria (independent, prompted, not reported, or not applicable). Finally, coders documented whether RIRD was implemented alone or in combination with additional procedures such as differential reinforcement or response cost. All variable definitions and decision rules appear in the Supplemental Materials to ensure transparency and reproducibility. Discrepancies in coding were resolved through discussion before proceeding to risk of bias and ethical coding.

### Risk of Bias Assessment

We conducted the risk of bias assessment using the protocol outlined by Reichow et al. (2018), which provides a structured method for evaluating the internal validity of single case research. Coders evaluated multiple variables including sequence generation, participant selection, blinding of participants and personnel, procedural fidelity, blinding of outcome assessment, selective outcome reporting, dependent variable reliability, and data sampling. These indicators were grouped into three bias categories: selection bias (sequence generation and participant selection), performance bias (blinding of participants and personnel and procedural fidelity), and detection bias (blinding of outcome assessment, selective reporting, dependent variable reliability, and data sampling). Each variable was classified as Low, High, or Unclear based on the definitions provided by Reichow et al. (2018). This framework allowed us to assess whether studies provided sufficient information to evaluate the appropriateness of participant selection, the consistency and accuracy of intervention delivery, and the reliability of outcome measurement, all of which align with the NIH ethical principle of fair subject selection.

### Ethical and Contextual Evaluation Framework

Ethical coding was conducted after WWC quality review and risk of bias assessment, using an ethical decision-making sequence informed by the NIH Guiding Principles for Ethical Research and the contextual fit framework described by Rojas et al. (2025). The NIH Guiding Principles for Ethical Research emphasize core considerations including social and clinical value, scientific validity, fair participant selection, favorable risk benefit balance, and respect for participants. These principles informed the organization of ethical coding into five domains reflecting whether RIRD was (a) contextually justified based on identified need, (b) supported by methodologically sound evidence, (c) implemented with adequate procedural integrity, (d) effective in producing meaningful behavior change, and (e) evaluated for social or clinical value through stakeholder input or acceptability measures. The contextual fit framework described by Rojas et al. (2025) further informed variable selection by highlighting the importance of aligning intervention decisions with environmental demands, stakeholder perspectives, and the functional impact of stereotypy on participation. Together, these frameworks provided a structured basis for examining how ethical considerations were operationalized and reported across the RIRD literature, rather than serving as a prescriptive or evaluative judgment of author intent or clinical appropriateness.

Variables were coded at the participant level and organized across five ethical domains. First, *need for treatment* was coded to determine whether authors provided a contextual rationale for targeting stereotypy, such as reported interference with learning, safety, social participation, or caregiver or stakeholder concern (e.g., contextual fit). Rationales were extracted verbatim when available to preserve author intent. Second, *scientific validity* was indexed using the WWC design ratings already assigned during the quality review, consistent with the NIH principle that interventions should be grounded in scientifically credible evidence. Only participants from studies meeting WWC design standards with or without reservations were retained for ethical analysis and descriptive synthesis. Third, *performance bias* was examined using the procedural fidelity indicators from the risk of bias assessment, including whether studies reported fidelity data, how fidelity was measured, and whether the integrity of RIRD implementation was monitored across sessions or implementers. Fourth, *efficacy of the intervention* was coded based on evidence of behavior change following RIRD, drawing from the WWC evidence analysis ratings. Finally, *social and clinical value* was coded by identifying whether studies reported social validity data, stakeholder evaluations, or acceptability outcomes. Together, these codes allowed us to evaluate not only the methodological rigor of the RIRD literature but also the ethical appropriateness of its application across participants and contexts.

### Descriptive Synthesis Procedures

Descriptive synthesis was conducted using the set of variables extracted during data extraction and coded across the ethical domains described above. Only participants from studies that met WWC design standards with or without reservations were included in the synthesis. For each eligible participant, data were summarized across the categories of participant characteristics, contextual fit indicators, functional behavior assessment procedures, and RIRD intervention characteristics. Coded variables were organized according to the ethical decision-making sequence, beginning with need for treatment, followed by scientific validity, fair subject selection, intervention efficacy, and social and clinical value. This structure allowed us to examine how methodological rigor, ethical justification, and contextual factors intersected across studies. Descriptive synthesis tables were generated to provide a participant level overview of these variables, and narrative summaries were used to integrate patterns across studies. Table 1 presents the full descriptive synthesis of all participants retained for analysis.

### Reliability

Interrater agreement (IRA) was assessed for all stages of the search process involving screening decisions because these judgments were binary (e.g., include or exclude). Agreement was defined as both raters making the same include or exclude decision, and disagreement as any mismatch. Percent agreement was calculated as agreements divided by agreements plus disagreements multiplied by 100. During the initial title and abstract screening, the second rater evaluated 50 of 149 records (34%), with 48 agreements (96%). For the full text review, the second rater evaluated 26 of 75 articles (35%), with complete agreement (100%). During the ancestral search screening, the second rater reviewed 13 of the 37 titles and abstracts (35%) with agreement for 11 of 13 records (85%). Across screening phases, the second rater evaluated at least 33% of records at each step (*M* = 39%; range = 35%–48%), and IRA averaged 92.7% (range = 85%–100%). All disagreements were resolved by consensus before advancing to later stages.

Interrater reliability (IRR) was evaluated for all coding procedures in the review, including participant level descriptive synthesis variables, contextual fit indicators, RIRD procedural characteristics, WWC design standards, WWC evidence standards, and risk of bias items. Because these variables required multi-category coding, agreement was defined as both raters assigning the same code, and disagreement as any mismatch in coding. Percent agreement was calculated using the same formula as IRA. For WWC design standards, the second rater independently coded 139 of 277 cases (50.2%), resulting in 847 agreements out of 959 ratings (88.3%). For WWC evidence standards, the second rater coded 51 of 142 eligible cases (36%), with 715 agreements out of 738 ratings (97%). For descriptive synthesis and contextual coding during the ancestral search, the second rater coded 15 of 43 eligible articles across 51 coding categories, achieving 47 agreements (92%). Across all coding domains, IRR ranged from 88.3% to 97%. All discrepancies were discussed and resolved until complete consensus was reached.

## Results

### Publication Trend Analysis

**Figure 2.**
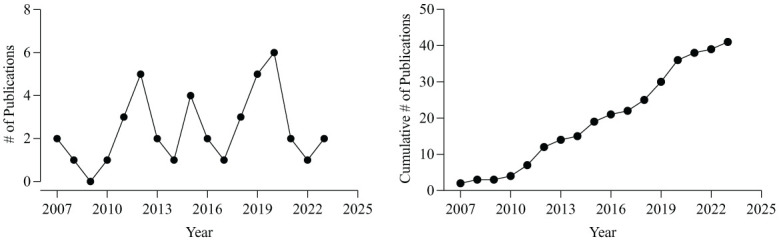
Annual (left) and cumulative (right) RIRD publication trends.

Figure 2 displays the annual (left) and cumulative (right) publication trends for RIRD-related studies over the past two decades. The annual trend illustrates high year-to-year variability, with noticeable spikes in research output approximately every 3 to 5 years, rather than a steady upward trajectory. These bursts may correspond to key conceptual or empirical developments in the field (see Discussion for further interpretation). In contrast, the cumulative trend reflects an overall increase in scholarly attention to RIRD. Together, these patterns help contextualize the trajectory of the literature reviewed in this study (Figure 2).

### Participant Characteristics

Table 1 summarizes participant characteristics for the 42 included studies, representing 109 unique participants. Participants ranged in age from 10 months to 30 years (*M* = 9.7 years), with the sample composed primarily of children and adolescents diagnosed with autism (*n* = 104; 95%). The sex was reported for all participants, with 84 males (77%) and 25 females (23%). Participant’s verbal repertoire was most often coded as yes (93%), that participants engaged in some form of verbal responses (*n* = 101), with only three reported as not having a verbal repertoire (Pastrana et al., 2013; Wunderlich & Vollmer, 2015), and five not being reported (Cook & Rapp, 2020; Meany-Daboul et al., 2007; Scully et al., 2023). Of the participants reported to have a verbal repertoire, communication modalities varied with 69 participants (68%) coded as using vocal verbal communication only, eight (8%) used sign or gesture only, four (4%) used speech-generating devices (SGD) only, two (2%) used picture exchange systems only, eight (8%) used a combination of two or more modalities, and 15 (15%) had insufficient information to code (e.g., not reported; NR). Verbal complexity was reported in 84 participants and majority (56%) had complex repertoires consisting of three or more-word phrases (*n* = 47), while 44% were coded as simple (*n* = 37). A total of 11 participants were noted to have a verbal repertoire, but the complexity was not mentioned or the manuscript had insufficient information for coding.

The topography of stereotypy was primarily vocal or motor. Most participants (64%) engaged in vocal stereotypy (*n* = 70), 25 (23%) engaged in motor stereotypy, nine (8%) engaged in combined vocal and motor stereotypy, and five (5%) engaged in other or less common repetitive behaviors such as public masturbation or object-based stereotypy. Functional behavior assessments were reported for most participants, although the methods differed widely. Most participants (68%) received a full experimental functional analysis (*n* = 74), 17 (16%) received an automatic reinforcement screening, two (2%) received only indirect assessments, and one (1%) received a descriptive assessment; 12 participants (11%) had no functional assessment procedures reported. Together, these characteristics provide a detailed picture of the populations, assessment practices, and behavioral profiles represented in the RIRD literature and serve as the foundation for the ethical and methodological analyses that follow.

### Contextual Fit

When assessing the contextual fit, we evaluated the need for treatment based on the authors’ report of a rationale for targeting vocal stereotypy other than the engagement of the behavior. Of the 109 participants included in this review, 76% (*n* = 83) reported a rationale for treatment. The rationale provided across studies included engaging in stereotypy that interfered with their participation in educational activities (e.g., Ahearn et al., 2007) or at high levels (e.g., Cassella et al., 2011), concern from the caregivers (e.g., Callahan et al., 2023) or clinical teams (e.g., Cividini-Motta et al., 2020), interference with skill acquisition (e.g., Barszcz et al., 2021), and/or social interactions (e.g., Gibney et al., 2020), and intrusive or disruptive in school or public settings (e.g., Chen & Traub, 2022). For the remaining 26 participants (24%), the authors did not provide a rationale for intervening on stereotypy. Interestingly, participants for whom no rationale was reported tended to appear in studies with limited assessment detail. In particular, these 26 cases were more likely to come from studies that used automatic-only screenings (*n* = 6; 23%), did not report FBA procedures at all (*n* = 6; 23%), or only received a descriptive assessment (*n* = 1; 4%), suggesting that the absence of contextual justification frequently co-occurred with minimal or incomplete functional assessment reporting.

### Scientific Validity: WWC Design Standards Results

Table 2 presents the results of the design standard evaluation. The final category for the overall design evaluation (e.g., “Met without Reservations”) is presented first in the table followed by the individual ratings for each design standard (e.g., rating for design standard #1 [#1] followed by the rating for design standard #2 [#2], etc.). The studies implemented RIRD yielded 277 individual cases. Of the 277 cases, 25 cases (9%) met design standards without reservations, 123 cases (44%) met design standards with reservations, and 129 cases (47%) did not meet design standards. Out of 123 cases that met design standards with reservations, 123 cases (*n* = 123; 100%) did not have a minimum of five data points to demonstrate effect (design standard “#4”). Out of 129 cases that did not meet design standards, 57 cases (*n* = 57; 44%) did not collect IOA on at least 20% of the data points in each condition (e.g., baseline, intervention; design standard “#2B”). 73 cases (*n* = 73; 57%) did not include at least three attempts to demonstrate an intervention effect at three different points in time or with three different phase repetitions (design standard “#3”). 10 cases (*n* = 10; 8%) did not have a minimum of three data points to demonstrate effect (design standard “#4”). The most common reason that cases did not meet design standards were that they did not have a minimum of three attempts to demonstrate effect (design standard “#3”).

**Table 2.** WWC Design Standards Results.

Article	Independent variable	Dependent variable	Case (# cases)	Design standard						
				#1 IV systematic manipulation	#2A IOA collected	#2B IOA 20% of phases	#2C IOA > minimum thresholds	#3 Attempts	#4 3–5 Data points	Overall
Met without Reservations										
Callahan et al. (2023)	RIRD	VS	Kevin (1)	1	1	1	1	1	2	**2**
	RIRD	VS	Joe (1)	1	1	1	1	1	2	**2**
	RIRD	VS	Nick (1)	1	1	1	1	1	2	**2**
Chen and Traub (2022)	RIRD	VS & AV	Eric (2)	1	1	1	1	1	2	**2**
Frewing et al. (2015)	RIRD	MS & VS	John (1)	1	1	1	1	1	2	**2**
Gibbs et al. (2018)	RIRD RIRD + MS	VS	Elizabeth (2)	1	1	1	1	1	2	**2**
A. F. Giles et al. (2012)	RD vs. RB	MS	Kelly (1)	1	1	1	1	1	2	**2**
	RD vs. RB	MS	Adam (1)	1	1	1	1	1	2	**2**
	RD vs. RB	MS	Spike (1)	1	1	1	1	1	2	**2**
A. Giles et al. (2018)	BST & RIRD	MS	Julie/Tim (1)	1	1	1	1	1	2	**2**
	BST & RIRD	MS	Kelly/James (1)	1	1	1	1	1	2	**2**
Martinez et al. (2016)	Signaled RIRD	VS & AV	Classroom (2)	1	1	1	1	1	2	**2**
	Signaled RIRD	VS & AV	Classroom Phase II (2)	1	1	1	1	1	2	**2**
Peters and Thompson (2013)	PP OC	MS & Engagement	Wes High-Preference Activity (2)	1	1	1	1	1	2	**2**
Toper-Korkmaz et al. (2018)	RIRD-1	VS & AV	Bryan (2)	1	1	1	1	1	2	**2**
Wells et al. (2016)	RIRD	VS	Roger (1)	1	1	1	1	1	2	**2**
Wunderlich and Vollmer (2015)	RIRD	VS	Kora (1)	1	1	1	1	1	2	**2**
	RIRD	MS & VS	Abby (2)	1	1	1	1	1	2	**2**
Met with Reservations										
Ahearn et al. (2007)	RI + RD	VS & AV	Mitch (2)	1	1	1	1	1	1	**1**
	RI + RD	VS & AV	Peter (2)	1	1	1	1	1	1	**1**
	RI + RD	VS & AV	Alice (2)	1	1	1	1	1	1	**1**
	RI + RD	VS & AV	Nicki (2)	1	1	1	1	1	1	**1**
Ahrens et al. (2011)	RIRD	MS, VS, AV	Bobby (4)	1	1	1	1	1	1	**1**
	RIRD	MS, VS, AV	Hal (4)	1	1	1	1	1	1	**1**
	RIRD	MS, VS, AV	Glen (4)	1	1	1	1	1	1	**1**
	RIRD	MS, VS, AV	David (4)	1	1	1	1	1	1	**1**
Brusa and Richman (2008)	RIRD	MS	Mark (1)	1	1	1	1	1	1	**1**
Cividini-Motta et al. (2019)	RIRD	MS, AV, Item Engagement	Ariel (3)	1	1	1	1	1	1	**1**
	RIRD	MS, AV, Item Engagement	Lewis (3)	1	1	1	1	1	1	**1**
	RIRD	MS, AV, Item Engagement	Sansita (3)	1	1	1	1	1	1	**1**
	RIRD + DRA	MS, AV, Item Engagement	Ariel (3)	1	1	1	1	1	1	**1**
	RIRD + DRA	MS, AV, Item Engagement	Lewis (3)	1	1	1	1	1	1	**1**
	RIRD + DRA	MS, AV, Item Engagement	Sansita (3)	1	1	1	1	1	1	**1**
Cividini-Motta et al. (2020)	RIRD	PM	Eddie (1)	1	1	1	1	1	1	**1**
	RIRD	PM	David (1)	1	1	1	1	1	1	**1**
	RIRD	PM	Carlos (1)	1	1	1	1	1	1	**1**
	RIRD	PM	Emily (1)							
Colón and Ahearn (2019)	RIRD	VS	Kent (1)	1	1	1	1	1	1	**1**
	RIRD	VS	Cora (1)	1	1	1	1	1	1	**1**
	RIRD	VS	Noah (1)	1	1	1	1	1	1	**1**
	RIRD	VS	Chad (1)	1	1	1	1	1	1	**1**
	RIRD	VS	Morris (1)	1	1	1	1	1	1	**1**
Dickman et al. (2012)	RIRD	VS & AV	Tobias (2)	1	1	1	1	1	1	**1**
	RIRD + DRI	VS & AV	Tobias (2)	1	1	1	1	1	1	**1**
A. Gibbs et al (2018)	RIRD MS + RIRD	VS	Matthew (2)	1	1	1	1	1	1	**1**
A. Giles et al. (2018)	BST & RIRD	MS	Kim/Daniel (1)	1	1	1	1	1	1	**1**
Love et al. (2012)	MS + RIRD	VS & AV	Ivan (2)	1	1	1	1	1	1	**1**
	MS + RIRD	AV	Troy (1)	1	1	1	1	1	1	**1**
Martinez et al. (2016)	Signaled RIRD	VS & AV	Classroom Generalization-listener task (2)	1	1	1	1	1	1	**1**
	Signaled RIRD	VS & AV	Classroom Generalization- speaker task (2)	1	1	1	1	1	1	**1**
McNamara and Cividini-Motta (2019)	RIRD RIRD + RC	VS	Karl (2)	1	1	1	1	1	1	**1**
	RIRD RIRD + RC	VS	Sammy (2)	1	1	1	1	1	1	**1**
	RIRD RIRD + RC	VS	Jon (2)	1	1	1	1	1	1	**1**
Meany-Daboul et al. (2007)	RIRD	VS & MS	Amy (3)	1	1	1	1	1	1	**1**
	RIRD	VS & MS	Daniel (3)	1	1	1	1	1	1	**1**
	RIRD	VS & MS	Beth (3)	1	1	1	1	1	1	**1**
Shawler and Miguel (2015)	VRIRD	VS & AV	Sally (2)	1	1	1	1	1	1	**1**
	VRIRD	VS & AV	Charlie (2)	1	1	1	1	1	1	**1**
	MRIRD	VS & AV	Charlie (2)	1	1	1	1	1	1	**1**
	MRIRD	VS & AV	Josh (2)	1	1	1	1	1	1	**1**
	VRIRD	VS & AV	Adam (2)	1	1	1	1	1	1	**1**
	VRIRD	VS & AV	Brayden (2)	1	1	1	1	1	1	**1**
	MRIRD	VS & AV	Brayden (2)	1	1	1	1	1	1	**1**
Steinhauser et al. (2021)	DRA + C-RD	VS	Andy (5)	1	1	1	1	1	1	**1**
	DRA + C-RD	MS	Hank (5)	1	1	1	1	1	1	**1**
	DRA + C-RD	VS & MS	Scott (4)	1	1	1	1	1	1	**1**
	DRA + C-RD	VS & MS	Seth (4)	1	1	1	1	1	1	**1**
	DRA + RIRD	VS	Sam (5)	1	1	1	1	1	1	**1**
Toper-Korkmaz et al. (2018)	RIRD-1	VS & AV	Nancy (2)	1	1	1	1	1	1	**1**
	RIRD-1	VS & AV	Areli (2)	1	1	1	1	1	1	**1**
Wunderlich and Vollmer (2015)	RIRD	VS	Ariel (1)	1	1	1	1	1	1	**1**
Did Not Meet Design Standards										
Barszcz et al. (2021)	Traditional & Modified RIRD	VS & Item engagement	Ben (4)	1	1	1	1	0	2	**0**
	Traditional & Modified RIRD	VS & Item engagement	Abby (4)	1	1	1	1	0	2	**0**
	Traditional & Modified RIRD	VS & Item engagement	Carol (4)	1	1	1	1	0	2	**0**
	Traditional & Modified RIRD	VS & Item engagement	David (4)	1	1	1	1	0	2	**0**
Carroll and Kodak (2014)	RIRD	VS	Will (6)	1	1	0	1	1	2	**0**
	RIRD	VS	Parker (6)	1	1	0	1	1	2	**0**
Cassella et al. (2011)	RIRD	VS	Adam (1)	1	1	0	1	1	2	**0**
	RIRD	VS	Chris (1)	1	1	0	1	1	2	**0**
Colón et al. (2012)	RIRD	VS & AV	Anna (2)	1	1	1	1	0	2	**0**
	RIRD	VS & AV	Jeff (2)	1	1	1	1	0	2	**0**
	RIRD	VS & AV	Parker (2)	1	1	1	1	0	2	**0**
Colón and Ahearn (2019)	RIRD	VS	Cora (1)	1	1	1	1	0	2	**0**
	RIRD	VS	Chad (1)	1	1	1	1	0	2	**0**
	RIRD	VS	Morris (1)	1	1	1	1	0	2	**0**
Cook and Rapp (2020)	PP OC		Sam (1)	1	1	0	1	1	2	**0**
	PP OC	MS, VS, & correct response	Sam (3)	1	1	0	1	1	2	**0**
DeRosa et al. (2019)	RIRD	MS	Zane (2)	0	1	0	1	1	1	**0**
	RIRD	MS	Richard (2)	1	1	0	1	1	1	**0**
	RIRD	MS	Caden (2)	1	1	0	1	0	2	**0**
Falligant and Dommestrup (2020)	RIRD + Stimulus Control	MS	David (1)	1	1	0	1	1	1	**0**
Gauthier et al. (2020)	RIRD	VS	Steve (2)	1	1	0	1	1	1	**0**
	RIRD	VS	Matt (2)	1	1	0	1	1	2	**0**
	RIRD	VS	Mary (2)	1	1	0	1	1	1	**0**
	RIRD	VS	Ben (2)	1	1	0	1	1	1	**0**
Gibney et al. (2020)	RIRD	VS & AV	Kallum (2)	1	1	1	1	0	1	**0**
	RIRD	VS & AV	Ben (2)	1	1	1	1	0	1	**0**
	RIRD	VS & AV	Harriett (2)	1	1	1	1	0	1	**0**
	RIRD	VS & AV	Andy (2)	1	1	1	1	0		**0**
Gould et al. (2019)	RIRD	VS & AV	David (2)	1	1	0	1	1	1	**0**
Love et al. (2012)	RIRD	VS & AV	Ivan (2)	1	1	1	1	0	1	**0**
	RIRD MS + RIRD	VS & AV VS	Troy (3)	1	1	1	1	0	1	**0**
Miguel et al. (2009)	RIRD + Sertraline	VS & AV	James (2)	1	1	0	1	1	1	**0**
	RIRD	VS & AV	James (2)	1	1	0	1	1	1	**0**
Pastrana et al. (2013)	RIRD	MS & VS	Emmett (2)	1	1	0	1	1	2	**0**
	RIRD	MS & VS	Andrew (2)	1	1	0	1	1	2	**0**
Peters and Thompson (2013)	PP OC	MS & Engagement	Max Low-Preference Activity (2)	1	1	1	1	0	2	**0**
	PP OC	MS & Engagement	Wes High-Preference Activity (2)	1	1	1	1	0	2	**0**
Saini et al. (2015)	RIRD	MS	Fabian (2)	1	1	0	1	1	1	**0**
	RIRD	VS	Walter (2)	1	1	0	1	1	1	**0**
	RIRD	MS	Barry (2)	1	1	0	1	1	1	**0**
	RIRD	MS	Carlton (2)	1	1	0	1	1	1	**0**
Schumacher and Rapp (2011)	RIRD	VS	Miranda (1)	1	1	0	1	1	2	**0**
	RIRD	VS	Nick (1)	1	1	0	1	1	2	**0**
Scully et al. (2023)	RD	MS	Repetitive body movement (2)	1	1	1	1	0	1	**0**
	RD	MS	Prolonged visual inspection (2)	1	1	1	1	0	0	**0**
Shawler et al. (2020)	RIRD	VS & AV	Paul (2)	1	1	0	1	0	1	**0**
	RIRD	VS & AV	Jane (2)	1	1	0	1	0	2	**0**
Shawler and Miguel (2015)	MRIRID	VS	Sally (2)	1	1	1	1	0	1	**0**
	VRIRD	VS	Josh (2)	1	1	1	1	0	1	**0**
	MRIRD	VS	Adam (2)	1	1	1	1	0	1	**0**
Sivaraman and Rapp (2020)	RIRD	VS & AV	Arun – Park (2)	1	1	1	1	1	0	**0**
	RIRD	VS & AV	Jason – Park (2)	1	1	1	1	1	0	**0**
	RIRD	VS & AV	Arun – Clinic (2)	1	1	1	1	1	0	**0**
	RIRD	VS & AV	Jason – Clinic (2)	1	1	1	1	1	0	**0**
Sloman et al. (2017)	Signaled RIRD	VS	Morning group (1)	1	1	1	1	0	1	**0**
	Signaled RIRD	VS	Independent activities (1)	1	1	1	1	0	2	**0**
	Intermittent RIRD	VS	Deskwork (1)	1	1	1	1	0	2	**0**
	Intermittent RIRD	VS	Community (1)	1	1	1	1	0	1	**0**
Steinhauser et al. (2021)	DRA + C-RD	VS	Sam (5)	1	1	1	1	0	2	**0**
Toper-Korkmaz et al. (2018)	RIRD-3	VS & AV	Nancy (2)	1	1	1	1	0	1	**0**
	RIRD-3	VS & AV	Bryan (2)	1	1	1	1	0	1	**0**
	RIRD-3	VS & AV	Areli (2)	1	1	1	1	0	1	**0**

*Note.* IV = independent variable; IOA = interobserver agreement; RIRD = response interruption and redirection; MS = motor stereotypy; VS = vocal stereotypy; AV = appropriate vocalization; RIRD + MS = response interruption and redirection + matched stimulation; BST = behavior skills training; PP OC = positive practice overcorrection; DRA + C-RD = differential reinforcement of alternative behavior + context-specific redirection; RI + RD = response interruption + redirection; RD = redirection; RB = response blocking; DRA = differential reinforcement of alternative behavior; DRI = differential reinforcement of incompatible behavior; RC = response cost; PM = public masturbation. Bolded numbers designate the overall design standard score.

#### Design Standards Given Experimental Design

Regarding the design standards given the experimental design, 192 of 277 cases (69%) employed a reversal design, 35 cases (13%) employed a multiple baseline (MBL) design, 16 cases (6%) employed an alternating treatments design (ATD), and 34 cases (12%) employed an AB design. Of the 35 cases utilizing an MBL design, 6 cases (17%) included 3 panels, whereas the remaining 29 cases (83%) included 2 panels. All cases that failed to meet design standards due to not including at least three attempts to demonstrate an intervention effect (design standard “#3”) were either using MBL designs with only two panels or AB designs. In contrast, cases using reversal or ATD designs failed to meet design standards due to IOA not being collected on at least 20% of data points in each condition (design standard “#2B”) or due to the inclusion of fewer than three data points (design standard “#4”).

#### Design Standards Given Intervention

Regarding design standards given the intervention, 104 of the 228 standalone RIRD cases (46%) met WWC design standards with or without reservations, while 124 cases (54%) did not. In contrast, when RIRD was combined with other procedures, 40 of 49 cases (82%) met design standards, and only 9 cases (18%) did not. This pattern suggests that studies incorporating RIRD within multicomponent or combined interventions were more likely to meet methodological rigor benchmarks than studies evaluating RIRD in isolation.

### Risk of Bias Assessment Outcomes

**Figure 3.**
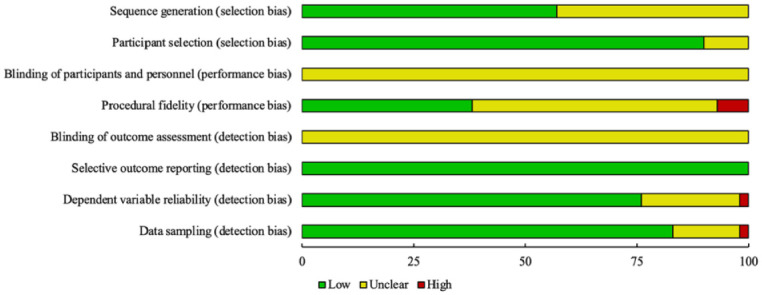
Risk of bias assessment summary plot.

Across the 42 included studies, patterns in risk of bias reflected both strengths and expected limitations of single-case research. Selection bias was generally low, with 24 studies (57%) providing sufficient information about sequence generation and 38 studies (90%) clearly describing participant selection procedures. In contrast, both blinding of participants and personnel and blinding of outcome assessors were uniformly coded as unclear (100% of studies), likely because these procedures are rarely feasible in single-case experimental designs rather than due to identifiable methodological flaws (Figure 3).

In contrast, performance bias, indexed through procedural fidelity, showed more variability. Specifically, only 16 studies (38%) reported fidelity at levels that met criteria for low risk, 3 studies (7%) reported concerns that constituted high risk, and 23 (55%) provided insufficient information to determine fidelity. Detection bias indicators were mixed. Selective outcome reporting was consistently low risk (42 of 42 studies, 100%), whereas dependent variable reliability was coded as low risk for 32 studies (76%), unclear for 9 (21%), and high for 1 (2%). Data sampling practices were generally adequate, with 35 studies (83%) meeting low-risk criteria, 6 (14%) rated as unclear, and 1 (2%) rated high risk. Together, these patterns suggest that while single case design-specific constraints inherently limit blinding, the more substantive concerns relate to incomplete reporting of fidelity and reliability procedures, both of which are common expectations in single-case research.

### Efficacy of Intervention: WWC Evidence Analysis Results

Across the 144 cases eligible for WWC evidence evaluation (i.e., those that met design standards with or without reservations), only 12% demonstrated Strong Evidence (*n* = 18), with a larger proportion (*n* = 79; 55%) scoring as Moderate Evidence, and the remaining cases (*n* = 47; 33%) classified as No Evidence of a functional relation. Based on the WWC scoring rubric, Strong Evidence was assigned to cases that met all evidence criteria, including clear demonstrations of effect, absence of non-effects, and a design rating of Meets Standards. A total of 18 cases met this threshold, representing the most methodologically robust demonstrations of RIRD effectiveness. An additional 79 cases were coded as Moderate Evidence, indicating that although a functional relation was suggested, at least one evidence criterion (for example, consistency of effect or the presence of a non-effect) reduced confidence in the causal interpretation. The remaining 47 cases were categorized as No Evidence, typically due to inconsistent demonstrations of effect, overlapping data patterns, or insufficient phase contrasts (Table 3).

**Table 3.** WWC Evidence Analysis Results.

Article	Independent variable	Dependent variable	Case (# cases)	Evidence standard					
				# Attempts	# Effects	Overall effect	Overall non-effect	Final design standard	Final evidence
Strong Evidence									
Callahan et al. (2023)	RIRD	Stereotypy	Kevin (1)	3	3	2	2	2	2
	RIRD	Stereotypy	Joe (1)	3	3	2	2	2	2
	RIRD	Stereotypy	Nick (1)	3	3	2	2	2	2
Chen and Traub (2022)	RIRD	VS	Eric (1)	3	3	2	2	2	2
Cividini-Motta et al. (2019)	RIRD	Stereotypy	Sansita (1)	3	3	2	2	1	2
	RIRD + DRA	Stereotypy	Ariel (1)	3	3	2	2	1	2
	RIRD + DRA	Stereotypy	Lewis (1)	3	3	2	2	1	2
	RIRD + DRA	Stereotypy	Sansita (1)	3	3	2	2	1	2
Gibbs et al. (2018)	RIRD	VS	Elizabeth (1)	3	3	2	2	2	2
	RIRD + MS	VS	Elizabeth (1)	3	3	2	2	2	2
A. F. Giles et al. (2012)	RD vs. RB	MV	Kelly (1)	3	3	2	2	2	2
	RD vs. RB	MV	Adam (1)	3	3	2	2	2	2
	RD vs. RB	MV	Spike (1)	3	3	2	2	2	2
Martinez et al. (2016)	Signaled RIRD	VS	Combined treatment settings (2)	5	5	2	2	2	2
	Signaled RIRD	VS listener tasks	Classroom (2)	3	5	1	2	2	2
	Signaled RIRD	AV listener tasks	Classroom (2)	3	3	2	2	2	2
Steinhauser et al. (2021)	RIRD	VS	Jane (1)	5	5	2	2	1	2
Moderate Evidence									
Ahearn et al. (2007)	RIRD	VS & AV	Mitch (2)	3	3	1	2	1	1
	RIRD	VS & AV	Peter (2)	3	3	1	2	1	1
	RIRD	VS & AV	Alice (2)	3	3	1	2	1	1
	RIRD	VS & AV	Nicki (2)	3	3	1	2	1	1
Ahrens et al. (2011)	Motor RIRD, Vocal RIRD	Stereotypy & AV	Bobby (4)	3	3	1	2	1	1
	Motor RIRD, Vocal RIRD	Stereotypy & AV	Hal (4)	5	5	1	2	1	1
	Motor RIRD, Vocal RIRD	Stereotypy	Glen (1)	3	3	1	2	1	1
Cividini-Motta et al. (2019)	RIRD	Stereotypy	Ariel (1)	3	3	1	2	1	1
	RIRD	Stereotypy	Lewis (1)	3	3	1	2	1	1
Cividini-Motta et al. (2020)	RIRD	PM	Eddie (1)	3	3	1	2	1	1
	RIRD	PM	David (1)	3	3	1	2	1	1
	RIRD	PM	Carlos (1)	3	3	1	2	1	1
	RIRD	PM	Emily (1)	3	3	1	2	1	1
Colón and Ahearn (2019)	RIRD duration	VS	Kent (1)	3	3	1	2	1	1
	RIRD duration	VS	Cora (1)	3	3	1	2	1	1
	RIRD duration	VS	Noah (1)	3	3	1	2	1	1
	RIRD duration	VS	Chad (1)	3	3	1	2	1	1
	RIRD duration	VS	Morris (1)	3	3	1	2	1	1
Dickman et al. (2012)	RIRD	VS	Tobias (1)	3	3	1	2	1	1
Gibbs et al. (2018)		VS	Matthew (1)	3	3	1	2	1	1
		On-task	Matthew (1)	3	3	1	2	1	1
McNamara and Cividini-Motta (2019)	RIRD, RIRD + RC	VS	Karl (2)	3	3	1	2	1	1
	RIRD, RIRD + RC	VS	Sammy (2)	3	3	1	2	1	1
	RIRD, RIRD + RC	VS	Jon (2)	3	3	1	2	1	1
Meany-Daboul et al. (2007)	RIRD	Duration & MTS	Amy (2)	3	3	2	2	1	1
	RIRD	Duration, MTS, PIR	Daniel (3)	3	3	1	2	1	1
	RIRD	Duration, MTS, PIR	Beth (3)	3	3	2	2	1	1
Shawler and Miguel (2015)	VRIRD	VS	Sally (1)	3	3	2	2	1	1
	MRIRD	VS	Charlie (1)	3	3	2	2	1	1
	VRIRD	VS	Charlie (1)	3	3	2	2	1	1
	VRIRD	AV	Charlie (1)	3	3	2	2	1	1
	MRIRD	AV	Josh (1)	3	3	2	2	1	1
	VRIRD	VS	Adam (1)	3	3	2	2	1	1
	MRIRD	VS & AV	Brayden (2)	3	3	2	2	1	1
Steinhauser et al. (2021)	DRA + C-RD	Engagement & stereotypy	Andy (3)	3	3	1	2	1	1
	DRA + C-RD	Stereotypy	Hank (2)	3	3	1	2	1	1
	DRA + C-RD	Engagement & stereotypy	Scott (2)	4	4	1	2	1	1
	DRA + C-RD	Engagement & stereotypy	Scott (2)	3	3	1	2	1	1
	DRA + C-RD	Stereotypy	Seth (1)	3	3	1	2	1	1
	DRA + C-RD	Stereotypy	Seth (1)	4	4	1	2	1	1
	DRA + C-RD	Stereotypy	Sam (1)	3	3	1	2	1	1
	DRA + C-RD	DTT responses	Sam (1)	4	4	1	2	1	1
	DRA + C-RD	Stereotypy	Sam (1)	3	3	1	2	1	1
	RIRD	AV	Paul (1)	5	5	1	2	1	1
	RIRD	AV	Jane (1)	5	5	2	2	1	1
Toper-Korkmaz et al. (2018)	RIRD-1	VS	Nancy (1)	5	5	2	2	1	1
	RIRD-1	VS	Areli (1)	5	3	2	2	1	1
	RIRD-1	VS	Bryan (1)	5	3	2	1	2	1
Wells et al. (2016)	RIRD	VS	Roger (1)	3	3	1	2	2	1
Wunderlich and Vollmer (2015)	RIRD	VS	Ariel (1)	3	3	1	2	2	1
	RIRD	VS	Harold (1)	3	3	2	2	2	1
	RIRD	VS	Kora (1)	3	3	2	2	2	1
	RIRD	VS	Abby (1)	3	3	2	2	2	1
Did Not Meet Evidence Standards									
Ahrens et al. (2011)	Motor RIRD, Vocal RIRD	Vocal Stereotypy & Motor Stereotypy	Glen (3)	3	3	0	2	1	0
	Motor RIRD, Vocal RIRD	Stereotypy & AV	David (4)	3	3	0	2	1	0
Brusa and Richman (2008)			Mark (1)	2	2	0	0	1	0
Chen and Traub (2022)	RIRD	AV	Eric (1)	3	1	0	1	2	0
Cividini-Motta et al. (2019)	RIRD	Item Engagement & AV	Ariel (2)	3	3	1	0	1	0
	RIRD	Item Engagement	Lewis (1)	3	3	1	0	1	0
		AV	Lewis (1)	1	1	1	0	1	0
	RIRD	Item Engagement	Sansita (1)	3	3	1	0	1	0
		AV	Sansita (1)	3	3	2	0	1	0
	RIRD + DRA	Item Engagement & AV	Ariel (2)	3	1	0	0	1	0
	RIRD + DRA	Item Engagement & AV	Lewis (2)	3	1	0	0	1	0
	RIRD + DRA	Item Engagement & AV	Sansita (2)	3	1	0	0	1	0
Dickman et al. (2012)	RIRD	AV	Tobias (1)	3	3	2	1	1	0
Frewing et al. (2015)			John (1)	5	5	2	0	1	0
A. Giles et al. (2018)	RIRD	Stereotypy	Julie/Tim (1)	3	3	0	0	2	0
	RIRD	Stereotypy	Kim/Daniel (1)	3	3	0	0	2	0
	RIRD	Stereotypy	Kelly/James (1)	3	3	0	0	2	0
Martinez et al. (2016)	Signaled RIRD	VS speaker tasks	Classroom (1)	3	3	1	0	1	0
	Signaled RIRD	AV speaker tasks	Classroom (1)	3	3	2	0	1	0
Meany-Daboul et al. (2007)	RIRD	PIR	Amy (1)	3	1	0	1	1	0
Shawler and Miguel (2015)	VRIRD	AV	Sally (1)	3	0	0	1	1	0
	MRIRD	AV	Charlie (1)	3	2	0	1	1	0
	MRIRD	VS	Josh (1)	3	1	0	1	1	0
	VRIRD	AV	Adam (1)	3	0	0	1	1	0
	VRIRD	VS & AV	Brayden (2)	3	2	0	1	1	0
Steinhauser et al. (2021)	DRA + C-RD	DTT responses & IT responses	Andy (2)	3	3	1	0	1	0
	DRA + C-RD	Engagement, DTT responses, IT responses	Hank (3)	3	3	1	0	1	0
	DRA + C-RD	Engagement	Seth (1)	3	3	1	0	1	0
	DRA + C-RD	Engagement	Seth (1)	4	4	1	0	1	0
	DRA + C-RD	IT responses	Sam (1)	4	4	1	0	1	0
	DRA + C-RD	Engagement	Sam (1)	3	3	1	0	1	0
Toper-Korkmaz et al. (2018)	RIRD-1	AV	Nancy (1)	5	0	1	1	1	0
	RIRD-1	AV	Areli (1)	5	1	0	1	1	0
	RIRD-1	AV	Bryan (1)	5	1	0	1	1	0

*Note.* When multiple dependent variables appear grouped within a study in this table; each represents a separate case that received identical WWC coding. Dependent variables were not analyzed jointly when assigning evidence ratings. RIRD = response interruption and redirection; MS = motor stereotypy; VS = vocal stereotypy; AV = appropriate vocalization; RC = response cost; DRA + C-RD = differential reinforcement of alternative behavior + context-specific redirection; DTT = discrete trial training; IT = incidental teaching.

Patterns in evidence ratings were associated with specific study features. Cases receiving Strong Evidence tended to involve clear, stable baselines, well separated level and trend changes, and high within-phase consistency. In contrast, cases classified as No Evidence often reflected variable or unpredictable baselines, limited demonstrations of effect, or inconsistent outcomes across separate cases reported within the same study. Additionally, several cases failed to demonstrate a functional relation despite receiving Meets Standards design ratings, highlighting that passing design standards does not guarantee evidence of effect. These findings underscore the importance of evaluating both design quality and demonstrated outcomes when determining the empirical support for RIRD.

#### Evidence Standards Given Intervention

When examining evidence outcomes by intervention type, RIRD implemented alone produced Strong or Moderate Evidence in 76 of 108 cases (70%), whereas 32 cases (30%) did not meet evidence standards. When RIRD was combined with additional procedures, 21 of 36 cases (58%) met Strong or Moderate Evidence criteria, and 15 cases (42%) did not. These patterns indicate that both standalone RIRD and combined-treatment formats can produce functional relations, though standalone RIRD showed a slightly higher proportion of cases meeting evidence standards.

### Social and Clinical Value

Of the 42 included articles, 16 (38%) reported some form of social validity assessment. Most assessments (75%, *n* = 12) involved surveys completed by individuals who cared for, worked with, or implemented intervention procedures for the participants, including parents (e.g., Callahan et al., 2023), teachers and instructional staff (e.g., A. Giles et al., 2018), and direct treatment implementers (e.g., Sloman et al., 2017). One study conducted interviews with teachers and educational assistants rather than a survey (Wells et al., 2016). Three studies incorporated participant-based evaluation methods. Two used concurrent-chain preference assessments to compare RIRD with alternative intervention components (Cividini-Motta et al., 2019; A. F. Giles et al., 2012), and one measured physiological responding (heart rate) as an index of participant preference and comfort (Frewing et al., 2015). Across studies that evaluated acceptability, reported ratings were consistently favorable, with stakeholders endorsing RIRD as effective, feasible, or acceptable when implemented under the conditions described. Participant-centered measures provided more nuanced information, underscoring the importance of incorporating learner perspectives into evaluations of intervention value.

For the two articles that assessed social validity using concurrent-chains preference assessments, participant choices consistently favored RIRD-based conditions. In A. F. Giles et al. (2012), all three participants (100%) selected redirection over response blocking when allowed to choose between the two procedural options. Similarly, in Cividini-Motta et al. (2019), two of the three participants (66%) exclusively selected RIRD when choosing among RIRD, RIRD combined with DRA, and DRA alone. The remaining participant followed a similar pattern of responding, selecting RIRD + DRA in 83% of opportunities, RIRD alone in 40% of opportunities, and DRA alone in 25% of opportunities, indicating a relative preference for intervention packages that incorporated RIRD. For the remaining article that included a participant-based assessment (Frewing et al., 2015) heart-rate monitoring showed no increase during or immediately following the implementation of RIRD with stimulus control procedures. This pattern was interpreted as preliminary evidence that the participant did not experience physiological distress during the intervention. Collectively, these participant-level outcomes suggest that when social validity was measured directly with autistic individuals, responses tended to favor RIRD-based procedures and did not indicate aversive physiological effects.

### Ethical and Contextual Evaluation Results

The ethical and contextual evaluation included only the subset of studies that met two criteria: (a) at least one case in the study achieved a WWC design standard rating of Meets Standards or Meets Standards with Reservations, and (b) at least one case demonstrated Strong or Moderate Evidence during the WWC evidence analysis. Ethical and contextual evaluation was intentionally restricted to studies meeting minimum WWC design and evidence standards to avoid interpreting ethical considerations in the absence of a demonstrated functional relation. Accordingly, findings from the ethical synthesis should be interpreted as conditional on baseline evidentiary thresholds rather than representative of the full RIRD literature.

Eighteen studies met these criteria and were included in the ethical evaluation (see Table 4). When a study included multiple participants whose WWC ratings differed (e.g., Cividini-Motta et al., 2019; Gibbs et al., 2018; Steinhauser et al., 2021), the highest rating occurring within that study was used for classification purposes. For example, in Cividini-Motta et al. (2019), all participants met WWC design standards, but evidence ratings varied; Sansita demonstrated Strong Evidence, whereas Ariel and Lewis demonstrated Moderate Evidence. This approach allowed us to capture the strongest available evidence from each article while maintaining consistency with the inclusion requirement.

**Table 4.** Ethical and Contextual Evaluation Results.

Article	Need for treatment	Scientific validity	Performance bias	Efficacy of intervention	Social and clinical value
Callahan et al. (2023)	Yes	2	Unclear	2	Yes
Chen and Traub (2022)	Yes	2	Unclear	2	No
Cividini-Motta et al. (2019)	Yes (Lewis only)	1	Unclear	2	Yes
Cividini-Motta et al. (2020)	Yes	1	High	1	Yes
Gibbs et al. (2018)	Yes	2	Low	2	Yes
A. F. Giles et al. (2012)	No	2	Unclear	2	Yes
Martinez et al. (2016)	No	2	Low	2	No
Steinhauser et al. (2021)	Yes	1	Unclear	2	No
Ahearn et al. (2007)	Yes	1	Low	1	No
Ahrens et al. (2011)	Yes	1	Unclear	1	No
Colón and Ahearn (2019)	Yes	1	Low	1	No
Dickman et al. (2012)	No	1	Low	1	No
McNamara and Cividini-Motta (2019)	Yes	1	Low	1	Yes
Meany-Daboul et al. (2007)	No	1	Unclear	1	No
Shawler and Miguel (2015)	Yes	1	Low	1	Yes
Toper-Korkmaz et al. (2018)	Yes	1	Low	1	No
Wells et al. (2016)	Yes	2	Low	1	Yes
Wunderlich and Vollmer (2015)	Yes	2	Unclear	1	No

Across the 18 studies, most authors provided a clear rationale for the *need for treatment* (*n* = 15; 83%), typically citing interference with learning, participation, or social interaction (e.g., Ahearn et al., 2007; Cassella et al., 2011), or caregiver or clinician concern (e.g., Callahan et al., 2023; Cividini-Motta et al., 2020). *Scientific validity* was consistently high, given that all included studies had already passed WWC design standards screening. *Performance bias*, indexed through procedural fidelity reporting, showed more variability: nine studies (50%) included explicit fidelity data, generally reporting high levels of implementer accuracy, whereas nine studies (50%) provided no fidelity information. *Efficacy of intervention* outcomes aligned with the WWC evidence classifications used for table inclusion, with strong evidence represented across some articles (39%; *n* = 7 studies) and moderate evidence reflected in a larger subset (61%; *n* = 11 studies). Finally, social and clinical value was reported in eight of the 18 studies (44%), most frequently through stakeholder rating scales or acceptability questionnaires (e.g., Sloman et al., 2017; Wells et al., 2016).

Taken together, the ethical synthesis highlights notable strengths in scientific validity and treatment need across the most rigorous segment of the RIRD literature, while also identifying persistent gaps in performance integrity reporting and inconsistent inclusion of social validity measures. These patterns provide a contextual foundation for interpreting the quality and appropriateness of RIRD applications observed across the broader literature.

## Discussion

The present review provides the most comprehensive methodological and ethical evaluation of the RIRD literature to date, extending earlier syntheses (Ledford et al., 2023; Lydon et al., 2013; Spencer & Alkhanji, 2018) by applying What Works Clearinghouse design and evidence standards in conjunction with a structured ethical and contextual framework. Taken together, the findings highlight a central conclusion: RIRD can be effective, ethical, and contextually appropriate when implemented under the right conditions. These conditions include (a) a clear and defensible need for treatment, (b) confirmatory evidence that the target behavior is maintained by automatic reinforcement, (c) high quality single case design features, and (d) transparent reporting of procedural fidelity and social validity. Across studies that incorporated these foundational elements, evidence of behavior reduction was consistently stronger, contextual justification was clearer, and stakeholder acceptance was higher. Conversely, studies that omitted these elements were more vulnerable to ethical concerns and weaker methodological outcomes, which likely fuels some of the contemporary criticism directed toward punishment-based interventions.

These findings help reconcile ongoing tensions in the field. Critics of RIRD, particularly those informed by neurodiversity perspectives (Bottema-Beutel et al., 2021; Kapp, 2020), have raised legitimate concerns about historical uses of punishment-based procedures that were implemented without contextual justification, stakeholder input, or adequate safeguards. Our review supports these concerns. In the subset of studies that lacked rationales for treatment, omitted functional behavior assessments, or failed to report fidelity, the use of RIRD was difficult to justify. Importantly, this does not suggest that RIRD is inherently inappropriate. Instead, it suggests that RIRD becomes ethically problematic when implemented without the prerequisite assessments and reporting practices needed to ensure that the procedure aligns with learner needs, stakeholder values, and evidence-based safeguards. In this sense, critics are often responding to experiences in which those conditions were *not* met. When they *are* met, the evidence paints a very different picture.

Notably, this review underscores that punishment-based interventions have undergone a marked evolution in recent years. Ferrier et al. (2025) documented a shift toward integrating punishment components only when embedded within broader, context sensitive interventions, rather than relying on punishment alone. Our findings align with this trend: studies with stronger evidence frequently combined RIRD with reinforcement procedures, implemented mastered tasks to minimize learner effort, or incorporated strategies designed to promote dignity and autonomy. Similarly, this review complements emerging contextual fit literature (Rojas et al., 2025) by demonstrating that when researchers explicitly identify why stereotypy is being targeted, how it affects participation or learning, and whether caregivers or stakeholders support the intervention, RIRD is more clearly justified and better aligned with contemporary expectations for ethical and acceptable practice. At the same time, the present review applied a targeted subset of contextual fit indicators and did not systematically evaluate additional dimensions emphasized by Rojas and colleagues, such as efficiency, implementation burden, cultural relevance, or long-term sustainability. These dimensions remain conceptually important for evaluating the appropriateness of RIRD, particularly given the response contingent nature of the procedure, and represent important directions for future research seeking to more fully integrate contextual fit considerations into the evaluation and application of RIRD.

One of the most meaningful contributions of this review is the synthesis of social validity data, particularly from participant centered assessments. Although only a subset of studies collected social validity, the results were noteworthy: when participants were given the opportunity to choose between intervention options, they often chose RIRD. Findings from concurrent chains analyses (Cividini-Motta et al., 2019; A. F. Giles et al., 2012) provide rare but compelling evidence that individuals may prefer structured interruption and redirection over alternatives like response blocking or reinforcement alone. These findings add a new dimension to ongoing debates about autonomy in autism intervention. They suggest that, at least for some individuals, RIRD may be experienced as acceptable, predictable, or effective in helping them manage high intensity stereotypy. This challenges assumptions that RIRD is universally aversive and reinforces the importance of systematically measuring and reporting social validity, including participant voice whenever possible.

Methodologically, this review advances prior syntheses by applying the WWC standards, which revealed a persistent but addressable gap: many RIRD studies have been designed and implemented in ways that limit their evidentiary strength. Critically, this review found that procedural fidelity remains one of the most underreported dimensions, a finding consistent with Ferrier et al. (2025) and broader punishment literature. Given that RIRD relies on precise, contingent responding by interventionists, fidelity lapses can substantially alter treatment effects, learner experience, and the interpretability of outcomes. The scarcity of fidelity reporting should be interpreted as a methodological gap in the field, not as evidence of poor implementation by practitioners.

It follows that we must address several limitations of the current review that warrant discussion. First, the review included only peer reviewed published studies and did not incorporate gray literature. This likely contributed to the uniformly low ratings for selective outcome reporting in the risk of bias assessment, as publication pipelines naturally screen for studies with complete reporting. Second, although this review applied a comprehensive ethical and contextual framework, it did not evaluate all variables described by Rojas et al. (2025), nor did it integrate all coded RIRD procedural variations reported in the supplemental materials. Third, the heterogeneity of reporting across studies limited the extent to which certain patterns, such as differences in procedural fidelity or redirection task type, could be meaningfully compared. Finally, social validity measures were sparse and varied substantially in methodology, which constrains firm conclusions about stakeholder and participant acceptability.

Together, these findings highlight several opportunities for future research. First, studies should systematically embed contextual fit assessments, including explicit documentation of need for treatment, stakeholder values, and alternative intervention options. Second, researchers should prioritize complete reporting of procedural fidelity, with direct measures rather than indirect or anecdotal descriptions. Third, social validity should be expanded to include the voices of autistic individuals wherever feasible, with methodologies that allow for preference expression even among learners with limited communication. Fourth, future research should evaluate RIRD within broader intervention systems, aligning with recent calls to conceptualize stereotypy intervention as part of comprehensive, person centered supports rather than as isolated behavior reduction efforts.

Finally, the field must adopt a consistent expectation that punishment-based procedures are only appropriate when contextual need is established, reinforcement-based alternatives have been considered, and high-quality design safeguards are in place. Inconsistency in meeting these criteria not only undermines the evidence base but also intensifies mistrust among autistic advocates and the broader community. Meeting these standards cannot be an exception. It must become the norm. By establishing a clear framework for ethical and contextually informed implementation, this review aims to contribute to that shift and to support a more transparent, rigorous, and socially responsive future for research on RIRD.

## Supplemental Material

sj-docx-1-bmo-10.1177_01454455261434871 – Supplemental material for Response Interruption and Redirection for Stereotypy: A Quality Review and Ethical Considerations Supplemental material, sj-docx-1-bmo-10.1177_01454455261434871 for Response Interruption and Redirection for Stereotypy: A Quality Review and Ethical Considerations by Hannah MacNaul and Anh Nguyen in Behavior Modification

sj-docx-2-bmo-10.1177_01454455261434871 – Supplemental material for Response Interruption and Redirection for Stereotypy: A Quality Review and Ethical Considerations Supplemental material, sj-docx-2-bmo-10.1177_01454455261434871 for Response Interruption and Redirection for Stereotypy: A Quality Review and Ethical Considerations by Hannah MacNaul and Anh Nguyen in Behavior Modification

sj-pdf-3-bmo-10.1177_01454455261434871 – Supplemental material for Response Interruption and Redirection for Stereotypy: A Quality Review and Ethical Considerations Supplemental material, sj-pdf-3-bmo-10.1177_01454455261434871 for Response Interruption and Redirection for Stereotypy: A Quality Review and Ethical Considerations by Hannah MacNaul and Anh Nguyen in Behavior Modification
