# Supplementary material for: Response Interruption and Redirection for Stereotypy: A Quality Review and Ethical Considerations
Source: Behav Modif. 2026 Apr 29;50(4):310–56. doi: 10.1177/01454455261434871 (PMC13237215; doi:10.1177/01454455261434871)
Supplement: sj-docx-1-bmo-10.1177_01454455261434871 – Supplemental material for Response Interruption and Redirection for Stereotypy: A Quality Review and Ethical Considerations [file sj-docx-1-bmo-10.1177_01454455261434871.docx]

**Supplemental Materials Table 1 (S1)**

*Coding Definitions*

| Variable(s) | Key definition |
| --- | --- |
| *Participant Characteristics* |  |
| Verbal Repertoire | Yes = Authors report the participant engaged in verbal responses (vocal or nonvocal), involving contextual emission of full English word(s), and does not include a repertoire limited to echoics of sounds that are not words  No = Authors report the participant did not engage in verbal responses  NR = Not reported; no discussion of the participant’s verbal repertoire |
| Communication Modality | Vocal = Authors note vocal verbal behavior, speaking, or echoic repertoire or using “full sentences” without otherwise specifying a different modality of communication  Sound-generating = “Sound-generating device,” “AAC device,” “communication device,” or specific sound-generating communication app (e.g., proloquo2go, TouchChat)  Picture exchange = “Picture exchange,” “PECS” or description of communicating via non-sound-generating pictures/icons  Sign / Gesture = “Sign,” “ASL,” “modified signs,” gestures, or physically directing individuals  NR = Not reported; verbal repertoire noted but no mention of the participant’s communication modality  NA = Not applicable; no verbal repertoire |
| Verbal Complexity | Simple = 1-2 words verbalization (e.g., 1-word mands, 1-word tacts, 1-word echoics), Does not include repertoires with only echoics of sounds that are not words  Complex = 3+ word combinations  NR = Not reported; verbal repertoire noted but no mention of the complexity of the participant’s communication repertoire  NA = Not applicable; no verbal repertoire |
| Target Behavior | Vocal Stereotypy = Authors report target behavior as “vocal stereotypy” or description of target behavior can be defined as noncontextual and repetitive vocalization, including words, phrases, or other vocalizations (e.g., sounds) **Author report is prioritized in coding  Motor Stereotypy = Authors report target behavior as “motor stereotypy” or description of target behavior can be defined as noncontextual and repetitive motor movement with or without objects (e.g., spinning items, hand flapping, body rocking) **Author report is prioritized in coding  Public Masturbation = Authors report target behavior as “public masturbation” or description of target behavior can be defined as sexualized behavior outside the context of a private setting (e.g., hands in groin area, hands in pants) **Author report is prioritized in coding  Other = Target behavior that does not meet the definitions of vocal stereotypy, motor stereotypy, or public masturbation. (This does not include specific topographies that the authors use that meet the above definitions [e.g., hair twirling-->MS; babbling-->VS]) |
| Receptive Communication | Yes = Authors report participant has receptive skills/repertoire, including following instructions  No = Authors report no receptive skills/repertoire  NR = Not reported; no mention of the participant’s receptive skills or following instructions |
| Motor Imitation | Yes = Authors report participant has motor imitation skills/repertoire  No = Authors report no motor imitation skills/repertoire  NR = Not reported; no mention of the participant’s motor imitation skills/repertoire |
| Contextual Fit | Yes = Authors provided a rationale (e.g., parents report concern) for including participants beyond their engagement in stereotypic behavior  No = Authors did not provided a rationale for intervening on stereotypy |
| FBA | Indirect = Authors report interviews, checklists, stakeholder questionnaires, etc. (e.g., Motivation Assessment Scale [MAS], Motivation Analysis Rating Scale [MARS], Problem Behavior Questionnaire [PBQ], Functional Analysis Screening Tool [FAST], Questions About Behavioral Function [QABF])  Descriptive = Authors report direct observations in naturally occurring conditions (e.g., ABC data, scatterplots)  Automatic Screen = Authors report repeated no interaction, no consequence, or alone sessions support assertions of the function of the target behavior (including repeated no interaction baseline sessions)  Tested Social Function = Authors tested 1+ social function (i.e., function other than automatic) with programmed consequences contingent on the target behavior  NR = Not reported; authors reported function but no mention of functional assessment  NA = Not applicable; no discussion of target behavior function |
| RIRD | Vocal = RIRD demands required vocal responses  Motor = RIRD demands required motor responses |
| Social Validity | Yes = Authors reported that social validity assessment was conducted (e.g., survey, questionnaire, concurrent chain preference assessment)  No = Authors did not report any format of social validity assessment |
| *RIRD Characteristics* |  |
| RIRD Analysis Setting | Controlled = RIRD was evaluated in the absence of naturally occurring activities (e.g., in a research room, in classroom but outside of regular programming)  Naturalistic = RIRD was evaluated during naturally occurring activities typical for the participant (e.g., special education classroom activities) |
| Generalization Setting | Controlled = RIRD was evaluated in the absence of naturally occurring activities (e.g., in a research room, in classroom but outside of regular programming)  Naturalistic = RIRD was evaluated during naturally occurring activities typical for the participant (e.g., special education classroom activities)  NA = No generalization across settings |
| RIRD Implementer | Researcher = Reported that researcher, research assistant, or graduate student implemented RIRD  Clinician = Reported that non-researcher clinician or service provider implemented RIRD (e.g., BCBA)  Teacher = Reported that teacher, teacher aide/assistant, or classroom staff implemented RIRD  Caregiver = Reported caregiver, parent, or guardian implemented RIRD  Other = Reported implementer other than above listed implementers  NR = Not reported, no mention of implementer  Unclear = RIRD implementer is unclear given the procedural details (e.g., general reference to “therapist”) |
| Generalization Implementer | Researcher = Reported that researcher, research assistant, or graduate student implemented RIRD  Clinician = Reported that non-researcher clinician or service provider implemented RIRD (e.g., BCBA)  Teacher = Reported that teacher, teacher aide/assistant, or classroom staff implemented RIRD  Caregiver = Reported caregiver, parent, or guardian implemented RIRD  Other = Reported implementer other than above listed implementers  NR = Not reported, no mention of implementer  Unclear = RIRD implementer is unclear given the procedural details (e.g., general reference to “therapist”)  NA = No generalization across implementers |
| Topography of RIRD Tasks | Vocal = RIRD demands required vocal responses  Motor = RIRD demands required motor responses |
| Type of Tasks | Mastered = Authors report that participants usually complied with the RIRD demands without requiring prompts (sometimes noted with a pre-assessment to identify RIRD demands)  Non-Mastered = Authors report unreliable responding to RIRD demands or RIRD involving the training of new non-mastered skills  NR = Not reported; no mention of the type of task |
| Redirection Topography | Verbal Redirection = Initial RIRD demand presented by implementer consisted of verbal redirection (i.e., prompt; e.g., verbal directive to play with toys)  Physical Redirection = Initial RIRD demand presented by implementer consisted of using physical redirection (i.e., prompt; e.g., physical guidance to play with toys)  NR = Not reported; no mention of topography of implementer’s initial RIRD prompt/presentation |
| Prompting Procedure | No Prompting = Implementer did not prompt compliance with RIRD demands or moved on to the next demand following no/incorrect response  Representation = Implementer represented the initial redirection following no/incorrect response  Prompt Hierarchy = Implementer provided a progressive series of prompts to prompt compliance following no/incorrect response  Verbal Prompting = Implementer provided verbal prompts to prompt compliance following no/incorrect response  Physical Prompting = Implementer provided physical prompts to prompt compliance following no/incorrect response  Topography NR = Not reported; no mention of topography of implementer’s prompts when prompting compliance after initial RIRD demand |
| Termination Criteria | Independent = Independent responding to RIRD demands was required to terminate RIRD  Prompted = Prompted responding to RIRD demands counted towards the termination criteria  Both = Both independent and prompted responding to RIRD counted towards the termination criteria  NR = Not reported; no mention of whether compliance was required to terminate RIRD  NA = Not applicable; there was no opportunity for independent compliance with RIRD demands (e.g., immediate physical redirection) or RIRD termination was based on some other measure (e.g., duration) |
| *RoB Assessment* |  |
| Sequence Generation | Low = Researchers randomly allocate participants to all conditions (e.g., baseline, control, intervention), identify sessions during which experimental conditions commence, assign the sequence or ordering of conditions, or randomization is not consistent with the conceptual foundations (e.g., selecting a tier in a multiple baseline design to commence intervention because the data were stable in that tier and not the others)  Unclear = randomization did occur, but the randomization method was not described clearly  High = randomization is conceptually important but did not occur using an appropriate method or if randomization could have been used but was not used (e.g., all tiers were stable in a multiple baseline design) |
| Participant Selection | Low = participants were selected based clearly on described inclusion criteria, an FBA, direct observation, or other conceptually important evidence of their performance relative to the dependent variable  Unclear = it is difficult to discern if adequate levels of target behavior were present before the study started or if the inclusion criteria were vague  High = no inclusion criteria or an inadequate description of the behavior studied were provided for the participants |
| Blinding of participants and personnel | Low = there are explicit blinding procedures described for blinding personnel to the participants and conditions  Unclear = blinding of participants and key personnel was not reported or lacked necessary detail to determine if personnel were truly naïve to participants and experimental conditions  High = there was no or incomplete blinding of participants and key personnel or blinding of participants and key personnel was either not attempted, not possible, or attempted but the blinding was likely or reported to be broken |
| Procedural Fidelity | Low = all conditions were described with replicable precision and fidelity was measured and reported for at least 20% of sessions in each experimental condition with 80% or greater implementation across all measurements or fidelity greater than a pre-established criterion  Unclear = (a) at least one condition was not described with replicable precision, (b) fidelity was not reported for all experimental conditions, or (c) at least 20% of sessions in each condition reported mean fidelity below 80%  High = (a) fidelity was reported for fewer than 20% of sessions across all experimental conditions, (b) mean fidelity across conditions were reported to be lower than 80%, (c) procedural fidelity did not meet pre-established criterion/a, or (d) procedural fidelity was not reported |
| Blinding of Outcome Assessment | Low = blinding of outcome assessors was ensured through implementation of an explicit set of procedures. Ex. Blinding procedures might include ambiguous data file labeling and storage procedures, locked and different offices for data collectors and implementers  Unclear = there was not adequate information provided in the study report to determine if outcome assessors were blind or if the issue was not addressed (reported) in the study report  High = outcome assessors remained aware of the research purpose or condition (e.g., if the primary data collector also was the primary implementer, if an intervention is present or absent during certain conditions and outcome assessors are aware this difference is the independent variable) |
| Selective Outcome Reporting | Low = there were no missing outcome data for any participants or the missing outcome data were unlikely to be related to the outcome or the clinical significance on the conclusions regarding the presence of a functional relation  Unclear = there is insufficient reporting of attrition, missing data, or additional exclusions to permit judgement of low risk  High = if one or more participants did not complete the study, study authors did not report data for at least one participant or one outcome, and the missing outcome data likely relates to the true outcome or produces clinically relevant bias regarding conclusions of a functional relation |
| Dependent Variable Reliability | Low = appropriate measures to estimate interrater agreement were used and mean IOA was reported to be greater than or equal to 80% (greater than or equal to .60 kappa) for all calculations in at least 20% of sessions in each condition, outcomes, or participants  Unclear = IOA was not measured in at least 20% of sessions in each condition, or appropriate measures were not used  High = mean IOA was lower than 80% (.60 kappa) |
| Data Sampling | Low = when there were an adequate number of data points in each condition to establish the level, trend, and variability (stability) of the data and the study included an adequate number of demonstrations of effect to determine if experimental control and functional relation(s) were established based on the study design used  Unclear = when there were not an adequate number of data points in each condition, when data have significant variability and the variability precluded predictions of data patterns, or the study did not include an adequate number of demonstrations of effect to infer a functional relation  High = when there were not an adequate number of data points in each condition and the study did not include an adequate number of demonstrations of effect to establish a functional relation |
