# Supplementary material for: Response Interruption and Redirection for Stereotypy: A Quality Review and Ethical Considerations
Source: Behav Modif. 2026 Apr 29;50(4):310–56. doi: 10.1177/01454455261434871 (PMC13237215; doi:10.1177/01454455261434871)
Supplement: sj-docx-2-bmo-10.1177_01454455261434871 – Supplemental material for Response Interruption and Redirection for Stereotypy: A Quality Review and Ethical Considerations [file sj-docx-2-bmo-10.1177_01454455261434871.docx]

**Supplemental Materials Table 2 (S2)**

*RIRD characteristics*

| Article | Name | RIRD analysis setting | Generalization setting | RIRD Implementer | Generalization Implementer | Topography of RIRD Tasks | Type of Tasks | Redirection Topography | Prompting Procedure | Termination Criteria |
| --- | --- | --- | --- | --- | --- | --- | --- | --- | --- | --- |
|  |  |  |  |  |  |  |  |  |  |  |
| Ahearn et al. (2007) | Mitch | Controlled | NA | Teacher | NA | Vocal | Mastered | Verbal Redirection | No Prompting | Independent |
|  | Nicki | Controlled | Naturalistic | Teacher | Teacher | Vocal | Mastered | Verbal Redirection | No Prompting | Independent |
|  | Peter | Controlled | Naturalistic | Teacher | Teacher | Vocal | Mastered | Verbal Redirection | No Prompting | Independent |
|  | Alice | Controlled | Naturalistic | Teacher | Teacher | Vocal | Mastered | Verbal Redirection | No Prompting | Independent |
| Ahrens et al. (2011) | Hal | Controlled | NA | Unclear | NA | Vocal, Motor | Mastered | Verbal Redirection | Prompt Hierarchy | Both |
|  | Hal (experiment 3) | Controlled | NA | Unclear | NA | Vocal | Mastered | Verbal Redirection | Prompt Hierarchy | Both |
|  | Bobby | Naturalistic | NA | Unclear | NA | Vocal, Motor | Mastered | Verbal Redirection | Representation, Verbal Prompting | Both |
|  | David (experiment 2) | Controlled | NA | Unclear | NA | Vocal, Motor | Mastered | Verbal Redirection | Prompt Hierarchy | Both |
|  | Glen (experiment 2) | Controlled | NA | Unclear | NA | Vocal, Motor | Mastered | Verbal Redirection | Representation, Verbal Prompting | Both |
| Barscazc et al. (2021) | Abby | Controlled, Naturalistic | NA | Researcher | NA | Vocal, Motor | Mastered | Verbal Redirection | Representation, Prompt Hierarchy | Both |
|  | Ben | Controlled, Naturalistic | NA | Researcher | NA | Vocal, Motor | Mastered | Verbal Redirection | Representation, Prompt Hierarchy | Both |
|  | Carol | Controlled, Naturalistic | NA | Researcher | NA | Vocal, Motor | Mastered | Verbal Redirection | Representation, Prompt Hierarchy | Both |
|  | David | Controlled, Naturalistic | NA | Researcher | NA | Vocal, Motor | Mastered | Verbal Redirection | Representation, Prompt Hierarchy | Both |
| Brusa & Richman (2008) | Mark | Naturalistic | NA | Teacher | NA | Vocal | NR | Verbal Redirection | Verbal Prompting | NR |
| Callahan et al. (2023) | Kevin | Naturalistic | Naturalistic | Unclear | Parent/Teacher | Vocal, Motor | Non-Mastered | Verbal Redirection | No Prompting | NA |
|  | Joe | Naturalistic | Naturalistic | Unclear | Parent/Teacher | Vocal, Motor | Non-Mastered | Verbal Redirection | No Prompting | NA |
|  | Nick | Naturalistic | Naturalistic | Unclear | Parent/Teacher | Vocal, Motor | Non-Mastered | Verbal Redirection | No Prompting | NA |
| Carroll & Kodak (2014) | Parker | Controlled | NA | Unclear | NA | Motor | Mastered | Verbal Redirection | Prompt Hierarchy | Both |
|  | Parker (experiment 2) | Controlled | NA | Unclear | NA | Motor | Mastered | Verbal Redirection | Prompt Hierarchy | Both |
|  | Will | Controlled | NA | Unclear | NA | Motor | Mastered | Verbal Redirection | Prompt Hierarchy | Both |
|  | Will (experiment 2) | Controlled | NA | Unclear | NA | Motor | Mastered | Verbal Redirection | Prompt Hierarchy | Both |
| Cassella et al. (2011) | Adam | Naturalistic | Naturalistic | Researcher | Clinician | Motor | NR | Verbal Redirection | Prompt Hierarchy | Both |
|  | Chris | Naturalistic | Naturalistic | Researcher | Clinician | Motor | NR | Verbal Redirection | Prompt Hierarchy | Both |
| Chen & Traub (2022) | Eric | Naturalistic | NA | Unclear | NA | Motor | Mastered | Verbal Redirection | Prompt Hierarchy | NA |
| Cividini-Motta et al. (2019) | Ariel | Naturalistic | NA | Clinician | NA | Vocal, Motor | Mastered | Verbal Redirection | No Prompting | Independent |
|  | Sansita | Naturalistic | NA | Clinician | NA | Vocal, Motor | Mastered | Verbal Redirection | No Prompting | Independent |
|  | Lewis | Naturalistic | NA | Clinician | NA | Motor | Mastered | Verbal Redirection | No Prompting | Independent |
| Cividini-Motta et al. (2020) | Eddie | Controlled, Naturalistic | NA | Clinician | NA | Motor | NR | Verbal Redirection | Prompt Hierarchy | NR |
|  | David | Controlled, Naturalistic | NA | Clinician | NA | Motor | NR | Verbal Redirection | Prompt Hierarchy | NR |
|  | Carlos | Controlled, Naturalistic | NA | Clinician | NA | Motor | NR | Verbal Redirection | Prompt Hierarchy | NR |
|  | Emily | Controlled, Naturalistic | NA | Clinician | NA | Motor | NR | Verbal Redirection | Prompt Hierarchy | NR |
| Colon & Ahearn (2019) | Chad (experiment 2) | Controlled | NA | Clinician | NA | Vocal | NR | Verbal Redirection | No Prompting | Independent |
|  | Morris (experiment 2) | Controlled | NA | Clinician | NA | Vocal | NR | Verbal Redirection | No Prompting | Independent |
|  | Noah | Controlled | NA | Clinician | NA | Vocal | NR | Verbal Redirection | No Prompting | Independent |
|  | Cora | Controlled | NA | Clinician | NA | Vocal | NR | Verbal Redirection | No Prompting | Independent |
|  | Kent | Controlled | NA | Clinician | NA | Vocal | NR | Verbal Redirection | No Prompting | Independent |
| Colon et al. (2012) | Anna | Controlled | NA | Researcher | NA | Vocal | Mastered | Verbal Redirection | Verbal Prompting | Both |
|  | Parker | Controlled | NA | Researcher | NA | Vocal | Mastered | Verbal Redirection | Verbal Prompting | Both |
|  | Jeff | Controlled | NA | Researcher | NA | Vocal | Mastered | Verbal Redirection | Verbal Prompting | Both |
| Cook & Rapp (2020) | Sam (Motor PPOC only) | Controlled | NA | Unclear | NA | Motor | Non-Mastered | Physical Redirection | Physical Prompting | NA |
|  | Sam (Motor & Vocal PPOC) | Controlled | NA | Unclear | NA | Motor | Non-Mastered | Physical Redirection | Physical Prompting | NA |
| DeRosa et al. (2019) | Zane | Controlled | NA | Clinician | NA | Motor | NR | Verbal Redirection | Prompt Hierarchy | Both |
|  | Caden | Controlled | NA | Clinician | NA | Motor | NR | Verbal Redirection | Prompt Hierarchy | Both |
|  | Richard | Controlled | NA | Clinician | NA | Motor | NR | Verbal Redirection | Prompt Hierarchy | Both |
| Dickman et al. (2012) | Tobias | Naturalistic | NA | Researcher | NA | Vocal | Mastered | Verbal Redirection | No Prompting | Independent |
| Falligant & Dommestrup (2020) | David | Controlled | Controlled | Clinician | Caregiver | Motor | NR | Physical Redirection | Physical Prompting | NR |
| Frewing et al. (2015) | John | Controlled | Naturalistic | Unclear | Unclear | Vocal, Motor | Mastered | Verbal Redirection | Representation | NA |
| Gauthier et al. (2020) | Mary | Controlled | NA | Unclear | NA | Motor | NR | Verbal Redirection | Prompt Hierarchy | Both |
|  | Matt | Controlled | NA | Unclear | NA | Motor | NR | Verbal Redirection | Prompt Hierarchy | Both |
|  | Ben | Controlled | NA | Unclear | NA | Motor | NR | Verbal Redirection | Prompt Hierarchy | Both |
|  | Steve | Controlled | NA | Unclear | NA | Motor | NR | Verbal Redirection | Prompt Hierarchy | Both |
| Gibbs et al. (2018) | Elizabeth | Controlled | Controlled | Researcher | Caregiver | Vocal | Mastered | Verbal Redirection | Verbal Prompting | Independent |
|  | Matthew | Controlled | Naturalistic | Researcher | Caregiver | Vocal | Mastered | Verbal Redirection | Verbal Prompting | Independent |
| Gibney et al. (2020) | Ben | Controlled, Naturalistic | Naturalistic | Researcher | Teacher, Caregiver | Vocal | Mastered | Verbal Redirection | Representation | Independent |
|  | Andy | Controlled, Naturalistic | Naturalistic | Researcher | Teacher, Caregiver | Vocal | Mastered | Verbal Redirection | Representation | Independent |
|  | Kallum | Controlled, Naturalistic | Naturalistic | Researcher | Teacher, Caregiver | Vocal | Mastered | Verbal Redirection | Representation | Independent |
|  | Harriet | Controlled, Naturalistic | NA | Researcher | NA | Vocal | Mastered | Verbal Redirection | Representation | Independent |
| Giles et al. (2012) | Kelly | Controlled | NA | Unclear | NA | Motor | Non-Mastered | Verbal Redirection | Prompt Hierarchy | NR |
|  | Adam | Naturalistic | NA | Unclear | NA | Motor | Mastered | Verbal Redirection | Prompt Hierarchy | NR |
|  | Spike | Naturalistic | NA | Unclear | NA | Motor | Mastered | Verbal Redirection | Prompt Hierarchy | NR |
| Giles et al. (2018) | James | Naturalistic | NA | Teacher | NA | Motor | NR | Verbal Redirection | Prompt Hierarchy | Both |
|  | Tim | Naturalistic | NA | Teacher | NA | Motor | NR | Verbal Redirection | Prompt Hierarchy | Both |
|  | Daniel | Naturalistic | NA | Teacher | NA | Motor | NR | Verbal Redirection | Prompt Hierarchy | Both |
| Gould et al. (2019) | David | Naturalistic | Naturalistic | Unclear | Unclear | Motor | NR | Verbal Redirection | Physical Prompting | NR |
| Liu-Gitz & Banda (2010) | Dylan | Naturalistic | NA | Teacher | NA | Vocal | Mastered | Verbal Redirection | NR | NR |
| Love et al. (2012) | Ivan | Naturalistic | NA | Unclear | NA | Vocal | Mastered | Verbal Redirection | NR | Independent |
|  | Troy | Controlled | NA | Unclear | NA | Vocal | Mastered | Verbal Redirection | NR | Independent |
| Martinez et al. (2016) | Peter (experiment 1) | Controlled, Naturalistic | NA | Unclear | NA | Motor | NR | Verbal Redirection | Representation, Physical Prompting | Independent |
|  | Peter (experiment 2) | Naturalistic | NA | Unclear | NA | Motor | NR | Verbal Redirection | Representation, Physical Prompting | Independent |
| McNamara & Cividini-Motta (2019) | Karl | Naturalistic | NA | Unclear | NA | Vocal | Mastered | Verbal Redirection | Representation, Verbal Prompting | Both |
|  | Sammy | Naturalistic | NA | Unclear | NA | Vocal | Mastered | Verbal Redirection | Representation, Verbal Prompting | Both |
|  | Jon | Naturalistic | NA | Unclear | NA | Vocal | Mastered | Verbal Redirection | Representation, Verbal Prompting | Both |
| Meany-Daboul et al. (2007) | Amy | Controlled | NA | Unclear | NA | Vocal | NR | Verbal Redirection | NR | NR |
|  | Daniel | Controlled | NA | Unclear | NA | Vocal | NR | Verbal Redirection | NR | NR |
|  | Beth | Controlled | NA | Unclear | NA | Motor | NR | Verbal Redirection | NR | NR |
| Miguel et al. (2012) | James | Controlled | NA | Unclear | NA | Vocal | Mastered | Verbal Redirection | NR | NR |
| Pastrana et al. (2013) | Emmett | Naturalistic | NA | Researcher | NA | Motor | Non-Mastered | Verbal Redirection | Representation, Physical Prompting | Independent |
|  | Andrew | Naturalistic | NA | Researcher | NA | Motor | Mastered | Verbal Redirection | No Prompting | Independent |
| Peters & Thompson (2013) | Max | Controlled | NA | Unclear | NA | Motor | NR | Physical redirection | Physical Prompting | NR |
|  | Wes | Controlled | NA | Unclear | NA | Motor | NR | Physical redirection | Physical Prompting | NR |
|  | Brett | Controlled | NA | Unclear | NA | Motor | NR | Physical redirection | Physical Prompting | NR |
| Saini et al. (2015) | Fabian | Controlled | NA | Unclear | NA | Motor | Mastered | Verbal Redirection | Prompt Hierarchy, Physical Prompting | Both |
|  | Walter | Controlled | NA | Unclear | NA | Motor | Mastered | Verbal Redirection | Prompt Hierarchy, Physical Prompting | Both |
|  | Carlton | Controlled | NA | Unclear | NA | Motor | Mastered | Verbal Redirection | Prompt Hierarchy, Physical Prompting | Both |
|  | Barry | Controlled | NA | Unclear | NA | Motor | Mastered | Verbal Redirection | Prompt Hierarchy, Physical Prompting | Both |
| Schumacher & Rapp (2011) | Miranda | Controlled | NA | Unclear | NA | Vocal | Mastered | Verbal Redirection | Representation | NA |
|  | Nick | Controlled | NA | Unclear | NA | Vocal | Mastered | Verbal Redirection | Representation | NA |
| Scully et al. (2023) | Ben | Naturalistic | NA | Unclear | NA | Motor | NR | Physical Redirection | Representation | NR |
| Shawler et al. (2020) | Paul | Controlled | NA | Researcher | NA | Vocal | Mastered | Verbal Redirection | Representation | NR |
|  | Jane | Controlled | Controlled | Researcher | Researcher | Vocal | Mastered | Verbal Redirection | Representation | NR |
| Shawler & Miguel (2015) | Sally | Controlled | NA | Unclear | NA | Vocal, Motor | Mastered | Verbal Redirection | Representation, Verbal Prompting | Independent |
|  | Cahrlie | Controlled | NA | Unclear | NA | Vocal, Motor | Mastered | Verbal Redirection | Representation, Verbal Prompting | Independent |
|  | Josh | Controlled | NA | Unclear | NA | Vocal, Motor | Mastered | Verbal Redirection | Representation, Verbal Prompting | Independent |
|  | Adam | Controlled | NA | Unclear | NA | Vocal | Mastered | Verbal Redirection | Representation, Verbal Prompting | Independent |
|  | Brayden | Controlled | NA | Unclear | NA | Vocal, Motor | Mastered | Verbal Redirection | Representation, Verbal Prompting | Independent |
| Sivaraman & Rapp (2015) | Arun | Controlled | Naturalistic | Researcher | Researcher | Vocal | NR | Verbal Redirection | Representation | NR |
|  | Jason | Controlled | Naturalistic | Researcher | Researcher | Vocal | NR | Verbal Redirection | Representation | NR |
| Sloman et al. (2017) | Elliot | Naturalistic | NA | Teacher | NA | Vocal | Mastered | Verbal Redirection | NR | NR |
| Steinhauser et al. (2021) | Andy | Controlled | NA | Unclear | NA | Vocal, Motor | NR | Verbal Redirection, Physical Redirection | NR | NR |
|  | Hank | Controlled | NA | Unclear | NA | Motor | NR | Verbal Redirection | NR | NR |
|  | Scott | Controlled | NA | Unclear | NA | Vocal, Motor | NR | Verbal Redirection | NR | NR |
|  | Seth | Controlled | NA | Unclear | NA | Vocal, Motor | NR | Verbal Redirection, Physical Redirection | NR | NR |
|  | Sam | Controlled | NA | Unclear | NA | Vocal | NR | Physical Redirection | NR | NR |
| Toper-Korkmaz et al. (2018) | Nancy | Controlled | NA | Unclear | NA | Vocal | Mastered | Verbal Redirection | Representation, Verbal Prompting | Independent |
|  | Areli | Controlled | NA | Unclear | NA | Vocal | Mastered | Verbal Redirection | Representation, Verbal Prompting | Independent |
|  | Bryan | Controlled | NA | Unclear | NA | Vocal | Mastered | Verbal Redirection | Representation, Verbal Prompting | Independent |
| Wells et al. (2016) | Roger | Naturalistic | NA | Teacher | NA | Vocal | Mastered | Verbal Redirection | NR | NR |
| Wunderlich & Vollmer (2015) | Ariel | Controlled | NA | Unclear | NA | Motor | Mastered | Verbal Redirection | Prompt Hierarchy, Physical Prompting | Both |
|  | Harold | Controlled | NA | Unclear | NA | Motor | Mastered | Verbal Redirection | Prompt Hierarchy | Both |
|  | Kora | Controlled | NA | Unclear | NA | Motor | Mastered | Verbal Redirection | Prompt Hierarchy | Both |
|  | Abby | Controlled | NA | Unclear | NA | Vocal, Motor | Mastered | Verbal Redirection | Prompt Hierarchy | Both |
|  | Daisy | Controlled | NA | Unclear | NA | Vocal, Motor | Mastered | Verbal Redirection | Prompt Hierarchy | Both |
|  | Drake | Controlled | NA | Unclear | NA | Vocal, Motor | Mastered | Verbal Redirection | Prompt Hierarchy | Both |
|  | Camilla | Controlled | NA | Unclear | NA | Motor | Mastered | Verbal Redirection | Prompt Hierarchy | Both |

**Note.** RIRD = response interruption and redirection, NA = not applicable, NR = not reported
