# Supplementary figures and images for: Response Interruption and Redirection for Stereotypy: A Quality Review and Ethical Considerations
Source: Behav Modif. 2026 Apr 29;50(4):310–56. doi: 10.1177/01454455261434871 (PMC13237215; doi:10.1177/01454455261434871)

S3. Risk of Bias Summary Plot

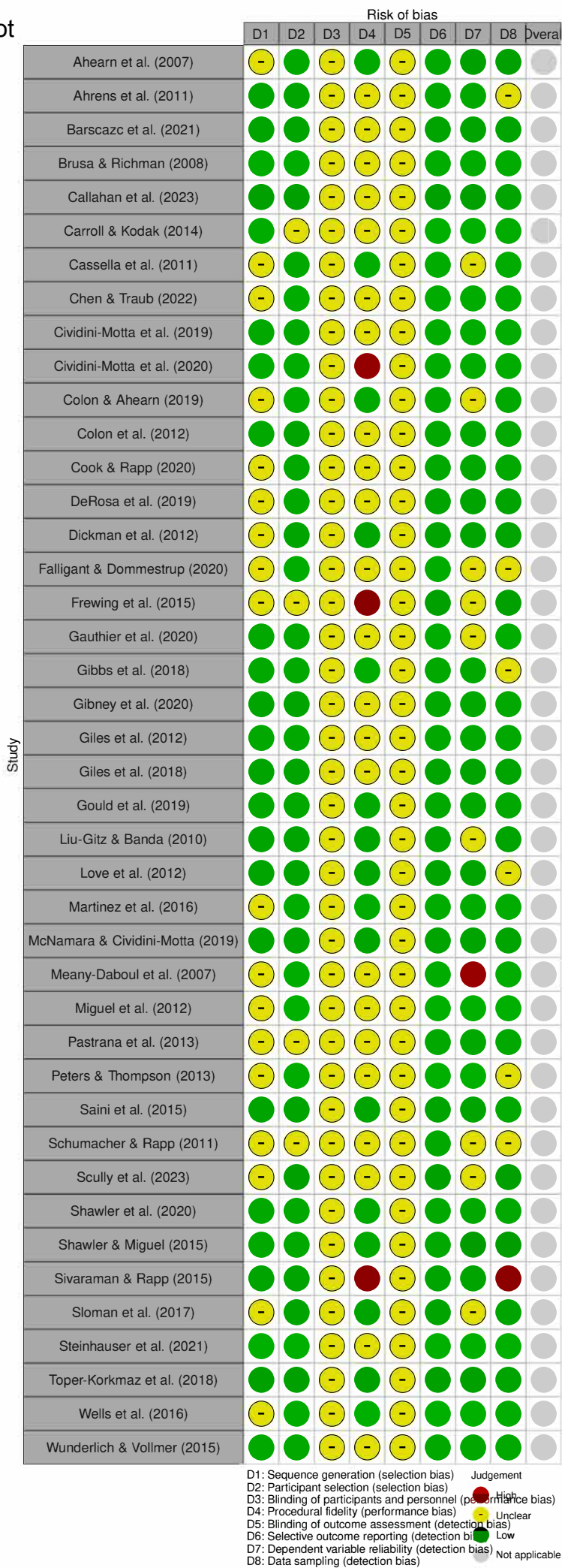

Supplement: sj-pdf-3-bmo-10.1177_01454455261434871 – Supplemental material for Response Interruption and Redirection for Stereotypy: A Quality Review and Ethical Considerations [file sj-pdf-3-bmo-10.1177_01454455261434871.pdf]
